# Fungal Diversity in Multiple Post-harvest Aged Red Pine Stumps and Their Potential Influence on Heterobasidion Root Rot in Managed Stands Across Minnesota

**DOI:** 10.3389/ffunb.2021.782181

**Published:** 2021-12-07

**Authors:** Eric C. Otto, Benjamin W. Held, Trevor J. Gould, Robert A. Blanchette

**Affiliations:** ^1^Division of Forestry, Minnesota Department of Natural Resources, Grand Rapids, MN, United States; ^2^Department of Plant Pathology, University of Minnesota, St. Paul, MN, United States; ^3^University of Minnesota Informatics Institute, University of Minnesota, St. Paul, MN, United States

**Keywords:** biological control, fungi, microbial ecology, *Phlebiopsis gigantea*, *Pinus resinosa*, root disease

## Abstract

Thinning operations that occur in managed red pine (*Pinus resinosa*) stands, create tree stumps that can serve as a habitat for fungi, especially *Heterobasidion irregulare*, the cause of a serious root disease. Different fungi can colonize stumps early and the community of fungi can change over time as initial fungal species become replaced. Samples were collected from both the native and non-native range of red pine from stumps that were cut at different time periods. Stumps that were harvested at 0–1, 2–3, 5–6, and 10–12 years before sampling were used to provide data on the diversity of fungi that colonize tree stumps and how these communities can change over time as well as how they influence colonization of *H. irregulare*. Traditional culturing methods and Illumina MiSeq sequencing were used to identify the fungi in the samples. Of particular interest was *Phlebiopsis gigantea*, which can colonize cut stumps and prevent *H. irregulare* from becoming established. Overall, *P. gigantea* was the most abundant fungus isolated and sequenced via Illumina MiSeq. Results show that *Phlebiopsis gigantea* was isolated from 90% of all stumps sampled for sites harvested within 3 years of sampling in the native range of red pine compared to 33% in the non-native range. For Illumina MiSeq, 5,940 total amplicon sequence variants (ASVs) were detected. *P. gigantea* represented 14% of the total reads and composed 19% of the reads in the native range and 8% in non-native range of red pine. Furthermore, *P. gigantea* represented 38% of the reads for stumps that were harvested within 3 years of sampling in the native range of red pine compared to 14% in the non-native range. These results help demonstrate that a higher amount of *P. gigantea* is present in the native range of red pine and could be acting as a native biological control agent. Additional fungi, including *Resinicium bicolor, Hypochnicium cremicolor, Leptographium* spp., and others identified at different cutting times are also discussed. Finally, different diversity indices revealed similar, but slightly higher diversity for southern sites via Shannon and Simpson Diversity indices. Beta diversity demonstrated a similar species composition in stumps harvested at different times with these stumps being grouped together based on harvesting years.

## Introduction

The thinning of red pine (*Pinus resinosa* Aiton) trees is a common management practice in pine plantations. It benefits the remaining pine trees by reducing the amount of competition for light, water, nutrients, and other resources, enabling faster and healthier growth. Overall, thinning will improve growing conditions, tree quality, and the economic value of the stand (Gilmore and Palik, 2005). Thinning operations will leave behind stumps that serve as a substrate for different microorganisms. Specifically, stumps are promptly available for fungal colonization. The phyla Ascomycota and Basidiomycota are primary colonizers of stumps and main decomposers in terrestrial ecosystems of the major wood components (cellulose, hemi-cellulose, and lignin) (Eriksson et al., 1990; Van Der Wal et al., 2015). The fungi can include white rot fungi that simultaneously or selectively decompose lignin, brown rot fungi that modify lignin during decomposition of cellulose and hemicellulose, and soft rot fungi that attack secondary cell wall components (Blanchette, 1995). The type of wood rot and fungal identity can also have major impacts to the decay rate of wood (Boddy, 2001; Schilling et al., 2015, 2020; Talbot et al., 2015).

The cut surface of a stump allows for rapid establishment of fungi via spores followed by colonization of the stump and roots (Rayner and Boddy, 1988; Pearce and Malajczuk, 1990). Some fungi that colonize a stump might also have been present before felling, such as fungi inhabiting the heartwood and latent invaders (Boddy and Heilmann-Clausen, 2008). Additionally, early establishment on stumps can be through a nearby mycelial colony or a network of mycelial cords (Boddy and Hiscox, 2017). After initial establishment, the fungal community will develop. Main driving forces have been established that influence fungal community development. These include, stress aggravation (worsening of abiotic environmental conditions), stress alleviation (improvement in abiotic conditions), and disturbance and combat (interspecific competition for space rather than directly for nutrients) (Cooke and Rayner, 1984; Rayner and Webber, 1984; Rayner and Boddy, 1988; Boddy, 2001; Heilmann-Clausen, 2001; Boddy and Heilmann-Clausen, 2008).

With these different forces underway, the fungal community in stumps will change over time. This can be due to two components of succession, primary and secondary resource capture. Primary resource capture involves the colonization of an unoccupied substrate and secondary resource capture is the colonization of a substrate previously colonized by other organisms (Rayner and Webber, 1984). A change in composition of the community is expected, but how that change takes place is dependent on certain variables and is important to understand. Early colonizers of stumps may also influence subsequent fungal communities that follow. These early colonizers can include opportunistic fungi that grow on accessible carbon sources and also have the capacity to grow on resins and extractives found in freshly cut wood (Van Der Wal et al., 2007; Hori et al., 2014).

The fungal pathogen, *Heterobasidion irregulare* Garbelotto & Otrosina, can be an early colonizer of stumps and was found in Minnesota in late 2014 (Blanchette et al., 2015). *Heterobasidion irregulare* is part of the species complex *H. annosum* sensu lato (s.l.). *Heterobasidion irregulare* causes Heterobasidion root disease (HRD) and is considered one of the most destructive disease agents of conifers in north temperate forests (Garbelotto and Gonthier, 2013). The primary infection is by airborne basidiospores produced by shelf-like basidiomes (Rishbeth, 1951). Freshly cut stumps that are left behind after thinning operations in managed forests serve as a substrate for airborne spores. After colonization, the pathogen moves into the root system and to adjacent trees by growing through the roots and connecting root contacts and grafts to adjacent living trees (Stenlid and Redfern, 1998). As the disease progresses on a site, circular infection centers are produced that continually expand producing circles of death (Stanosz et al., 2016). This study in addition to examining fungi present on stumps harvested at different times since sampling, was also done to survey for *H. irregulare* in Minnesota. *H. irregulare* can be a difficult fungus to culture and using high-throughput sequencing is an additional tool to help detect this pathogen in its early stages of colonization.

Other pioneer colonizing fungi can also become established on freshly cut stumps and may prevent *H. irregulare* from colonizing (Rishbeth, 1959). One species of interest is *Phlebiopsis gigantea*, which has previously been found to be abundant on stumps, dead and dying trees, and other woody debris in Minnesota (Otto et al., 2021). It has also been well studied as an effective biological control agent to use for HRD worldwide (Pratt et al., 2000; Nicolotti and Gonthier, 2005; Dumas and Laflamme, 2013; Terhonen et al., 2013; Oliva et al., 2017; Zaluma et al., 2021). If *P. gigantea* naturally colonizes stumps before *H. irregulare*, it could act as a native biological control agent. Over time these early colonizers, such as *P. gigantea*, are replaced by secondary decay species, which can then be replaced by more aggressive species and stress-tolerant species (Holmer and Stenlid, 1997; Boddy, 2001; Boddy and Heilmann-Clausen, 2008).

In the past, wood-decay fungi have often been evaluated by primarily observing fungal fruiting bodies (Yamashita et al., 2015). However, recent studies have revealed a rich diversity of fungi on stumps and coarse woody debris using mycelial extractions or molecular methods (Van Der Wal et al., 2015; Bonito et al., 2016; Kubart et al., 2016; Kaitera et al., 2019). Additionally, studies have shown differences in fungal species diversity and composition can be found when using fruiting bodies, culturing, or molecular techniques (Rajala et al., 2010, 2012; Ovaskainen et al., 2013; Bonito et al., 2016). The use of high-throughput sequencing has shown a much higher fungal diversity compared to sporocarp surveys, mycelial isolations, and earlier molecular methods (Ovaskainen et al., 2010, 2013; Kubartov et al., 2012; Kubart et al., 2016; Van der Wal et al., 2017).

This study reports results using a combination of traditional culturing methods and high-throughput sequencing. Sampling sites consisted of those in the native range of red pine and the non-native range within the state of Minnesota and these sites were harvested at different times. Overall, this resembles a chronosequence, which has been defined as a set of sites that have similar attributes, but represent different ages (Johnson and Miyanishi, 2021). Decay stages of I to V were also determined to obtain information on how much decay was in the stumps (Hottola and Siitonen, 2008). In addition, the presence of *H. irregulare* and *P. gigantea* and other fungi in cut stumps were investigated at different post-harvest times.

## Materials and Methods

### Study Sites and Sampling

Stumps were sampled from eight different locations in Minnesota (Table 1). Four sites were in the native range of red pine in northern Minnesota in Carlton County and four locations in the non-native range of red pine in southeastern Minnesota in Goodhue, Houston, Wabasha, and Winona Counties. The locations were chosen based on the year the stands were thinned in order to obtain a chronosequence of the fungi present in the stumps. The stands consisted of four groups: thinned 0–1, 3–4, 5–6, and 10–12 years before sampling. Stumps from these stands were referred to as fresh, young, old, and very old for sampling purposes. A total of 120 stumps were sampled with 60 stumps in the native and 60 in the non-native range of red pine. Stumps were randomly sampled. The average size of stumps sampled was 12.33 cm in height and 28.96 cm in diameter. A drill bit (approximately 1 cm in diameter and 9 cm in length) was used for sampling. Bark was removed and drilling was done around the stump at each cardinal direction. Samples were extracted horizontally, approximately 10 cm below the stump cut surface. Only sapwood was collected since most trees are harvested during thinning operations before extensive heartwood develops (Boddy and Heilmann-Clausen, 2008). Additionally, the focus of this study was just on fungi colonizing the sapwood. The drill bit was sterilized with 70% ethanol between each sampling and wood from the drilling was collected in sterile plastic bags. The wood shavings were combined from each of the four drill locations for each stump. The sterile plastic bags were kept cool while transported back to the University of Minnesota where samples were stored at −20°C until processing. Stumps were sampled from July to October of 2016.

**Table 1:** Locations of study sites where trees were cut during thinning leaving stumps of red pine.

**County**	**Town/Township**	**Years since thinning**	**Latitude**	**Longitude**	**No. of samples**	**Decay stage**
Carlton	Cloquet	<1	46° 41′ 25″ N	92° 33′ 03″ W	15	I
Goodhue	Hay Creek Twp.	<1	44° 31' 03″ N	92° 32′ 01″ W	15	I
Carlton	Cloquet	2	46° 42′ 46″ N	92° 29′ 07″ W	15	I-II
Wabasha	Greenfield Twp.	3	44° 19′ 48″ N	92° 03′ 17″ W	15	I-II
Carlton	Cloquet	5	46° 41′ 54″ N	92° 31′ 31″ W	15	III
Houston	Money Creek Twp.	6	43° 47′ 02″ N	91° 40′ 36″ W	15	III
Carlton	Cloquet	10	46° 41′ 30″ N	92° 33′ 31″ W	15	IV
Winona	Mount Vernon Twp.	12	44° 10′ 43” N	91° 54′ 24″ W	15	IV

*Trees were harvested at different times in the native (Carlton County) and non-native (other counties) range for red pine in Minnesota. The decay stage for each study site is also provided*.

### Isolating Fungi From Stumps

Under sterile conditions in the laboratory, the wood shavings were cut into small segments and placed onto the following types of growth media: 1.5% Difco malt extract agar (15 g of malt extract, 15 g of agar), a selective medium used to culture ophiostomatoid fungi that cause blue stain (20 g of malt extract, 15 g of agar with 0.1 g cycloheximide and 0.01 g of streptomycin sulfate added after autoclaving), and a semi-selective medium used to culture Basidiomycota that was slightly modified from Worrall (1991) (20 g of malt extract, 15 g of agar, 2 g of yeast extract, 0.06 g of benlate with 2 ml of lactic acid and 0.1 g of streptomycin sulfate added after autoclaving). The media used in this study have been previously shown to be successful in isolating fungi from different wood samples (Blanchette et al., 2016, 2021). Isolations were made from all 120 stump samples from the native and non-native range of red pine. Cultures were grown at room temperature (~20°C) and observed daily. Sub-cultures were made to obtain pure cultures and placed on the same media they were cultured on.

### DNA Extraction, PCR, and Sanger Sequencing of Isolations

After the pure cultures reached an adequate size, a cetyl trimethylammonium bromide (CTAB) extraction protocol was used to extract DNA (Blanchette et al., 2016). For PCR amplification, the internal transcribed spacer (ITS) region of ribosomal DNA was targeted by using the primers ITS1F/4 (Gardes and Bruns, 1993). PCR amplifications were prepared with 12.5 μl of GoTaq®, 9.5 μl of sterile water, 1 μl of each primer (0.5 μM), 0.5 μl BSA, and 1 μl of template DNA. A thermocycler with the following parameters was used: 94°C for 5 min, 35 cycles of 94°C for 1 min, 50°C for 1 min, 72°C for 1 min, and a final extension step of 5 min at 72°C. Visualization of PCR amplicons was completed on 0.08 and 0.05% agarose gels using SYBR green 1 prestain and transilluminated with a Dark Reader DR45 (Clare Chemical Research, Denver, Colorado). Sequencing was performed with the forward primer, ITS1F, using an ABI 3730xl DNA sequencer (Applied Biosystems, Foster City, CA, USA). Assembly of consensus sequences was done using Geneious 9.0 (Kearse et al., 2012) and compared to sequences in GenBank using BLASTn for identification.

*Leptographium* cultures had additional loci amplified for identification that included beta tubulin (TUB) and translation elongation factor-1 alpha (TEF-1α Primers used for TUB included T10 and Bt2b and EF2-F and EF2-R for TEF-1α Yin et al., 2019).

### DNA Extraction, PCR, and High-Throughput Sequencing of Wood Shavings

DNA was extracted directly from the stump wood shavings using the PowerPlant Pro DNA Isolation kit (MO BIO Laboratories, Carlsbad, California, USA) following the manufacturer's instructions. The presence of DNA was confirmed with PCR amplification targeting the ITS region of ribosomal DNA and using the visualization method as described previously. Samples were then prepared for submission to the University of Minnesota Genomics Center (UMGC). A total of 20 μl of DNA was used from each of the 120 samples. The DNA was pipetted into 96 well plates and submitted to be sequenced. The internal transcribed spacer 1 (ITS1) of the nuclear ribosomal DNA was used as a DNA barcode for molecular species identification (Monard et al., 2013; Mbareche et al., 2020). The high-throughput sequencing was conducted at UMGC with paired-end sequencing (2 × 300 bp) on an Illumina MiSeq system using the MiSeq Reagent Kit v3 (600 cycles) following the manufacturer's protocol.

### Analysis of Sequencing Data

Initial processing for removal of sequence adapters and primers was done using cutadapt (Martin, 2011). All further processing was performed using R (R Core Team, 2017). Removal, filtering, and trimming of low quality sequences and chimeric reads was done using DADA2 (Callahan et al., 2016). The remaining sequence data was used for error inference and the creation of an ASV table. Taxonomic identification of ASVs was done using IDTAXA in the decipher R package (Wright, 2016) using the current SILVA database (Quast et al., 2013). The analysis of ASV alpha diversity was done using the vegan package (Oksanen et al., 2020) for Shannon, Simpson, and inverse Simpson metrics and a CLR transform was used to calculate beta diversity. All plots were made with ggplot2 (Wickham, 2009) using color palette from wesanderson (GitHub, 2016).

## Results

### Isolates—Stump Samples

A total of 289 isolates were obtained from all eight locations with 178 Ascomycota, 83 Basidiomycota, 24 Mucormycota, and 4 Mortierellomycota. Table 2 displays abundant and relevant taxa isolated from stumps and Supplementary Table 1 displays all taxa isolated. These represented 69 different taxa from Ascomycota, 27 taxa from Basidiomycota, 7 taxa from Mucormycota, and 3 from Mortierellomycota. If the same taxon was isolated multiple times from the same stump, only one was reported. The most commonly isolated fungus was *Phlebiopsis gigantea* representing 13% of all isolated fungi and 46% of the Basidiomycota isolated. The second most commonly isolated Basidiomycota was *Pholiota spumosa* at 6% followed by *Irpex lacteus*, and *Sistotrema brinkmannii* at just 5% of the Basidiomycota isolates. The most commonly isolated Ascomycota was *Metapochonia bulbillosa* representing 13% of the isolates from Ascomycota. Other commonly isolated taxa from Ascomycota included *Scytalidium album* at 7% and *Ophiostomatales* sp. and *Mariannaea elegans* at 6%. Additionally, three *Leptographium* species, *L. lundbergii, L. procerum*, and *L. terebrantis* combined were isolated for a total of 6% for *Leptographium* spp.

**Table 2:** List of the most abundant fungal taxa obtained and number of isolations of taxa from stumps at all sites with native and non-native designating the range of red pine.

**Taxa**	**Native (0–1)**	**Non-native (0–1)**	**Native (2–3)**	**Non-native (2–3)**	**Native (5–6)**	**Non-native (5–6)**	**Native (10–12)**	**Non-native (10–12)**	**Total**	**GenBank accession #**
**Ascomycota**										
*Alternaria alternata*	2	1		1	1			2	7	OK173732
*Diplodia sapinea*		3						2	5	OK173752
*Epicoccum nigrum*	2					1			3	OK173753
*Hormonema macrosporum*	1		1		3				5	OK173756
*Leptographium lundbergii*	1								1	OK173765
*Leptographium procerum*								2	2	OK173766
*Leptographium terebrantis*			8						8	OK173767
*Mariannaea elegans*	1			1		2	1	5	10	OK173769
*Metapochonia bulbillosa*		2		3	3	6	1	8	23	OK173770
*Ophiostomatales* sp. SM13-21-21-3				3	4	4			11	OK173780
*Scytalidium album*	1	3		2	2		3	1	12	OK173788
*Trichoderma deliquescens*			4						4	OK173791
*Trichoderma harzianum*		2	1	3					6	OK173793
**Basidiomycota**										
*Irpex lacteus*						4			4	OK173807
*Phlebiopsis gigantea*	13	9	14	1	1				38	OK173819
*Pholiota spumosa*			1		1		3		5	OK173820
*Sistotrema brinkmannii*			1	1	1	1			4	OK173823
**Mucormycota**										
*Umbelopsis isabellina*	1		1		3		6	2	13	OK173830

*The numbers in parentheses indicate the years since the stumps were cut and sampling took place. Taxa were identified using ITS sequence comparisons to the best BLASTn match with the NCBI GenBank database. GenBank accession numbers for each taxa are also listed*.

For the Basidiomycota found at sites harvested 0–1 years before sampling, only four taxa were isolated with two from the northern location and three from the southern location. *P. gigantea* was the most abundant representing 81% of the total Basidiomycota isolates. The northern and southern locations were similar with *P. gigantea* being isolated 81% and 82% out of the Basidiomycota isolated for those locations. More importantly, *P. gigantea* was isolated from 13 of 15 stumps at the northern location and 9 of 15 stumps at the southern location. More Basidiomycota taxa were isolated from the northern and southern locations harvested 2–3 years before sampling with 11 different taxa, four from the northern location and nine from the southern. *Phlebiopsis gigantea* was the most abundant representing 58% of the Basidiomycota. At the northern location, 82% of the isolates were *P. gigantea* compared to 11% at the southern location. Additionally, *P. gigantea* was isolated out of 14 of 15 stumps at the northern location and 1 of 15 stumps at the southern location. The most Basidiomycota taxa were isolated from sites harvested 5–6 years before sampling with 17 different taxa, with 10 from the northern location and eight from the southern. The most abundant was *Irpex lacteus* at 17%, which was just isolated from the southern location. *Scytinostroma* sp. was the most abundant at the northern location, isolated at just 9%. The northern and southern locations harvested 10–12 years before sampling had five different Basidiomycota taxa with three at the northern and two at the southern location. *Pholiota spumosa* was the most commonly isolated at 43%, all at the northern location, but out of just seven total isolates.

For Ascomycota from sites harvested 0–1 years before sampling, 27 different taxa were isolated, with 14 from the northern location and 16 from the southern. *Diplodia sapinea* and *Scytalidium album* were the most abundant, both representing 7% of the total Ascomycota at these sites. A total of 28 different taxa were isolated from sites harvested 2–3 years before sampling, with the northern site having 13 taxa and the southern site having 16. The most abundant isolate was *Leptographium terebrantis* representing 16% of the Ascomycota isolates. All of the isolates were from the northern location. A total of 22 different taxa were isolated from sites harvested 5–6 years before sampling with the northern site having 12 taxa and the southern location with 13. The most abundant isolate was *Ophiostomatales* sp. representing 18% of the total Ascomycota isolates. The northern and southern locations harvested 10–12 years before sampling had 22 different taxa with 10 from the northern location and 15 from the southern. The most abundant isolates were *Metapochonia bulbillosa* representing 21% and *Mariannaea elegans* representing 14%. The majority of these isolates were isolated from the southern location.

### Amplicon Sequence Variants—Stump Samples

**Figure 1:**
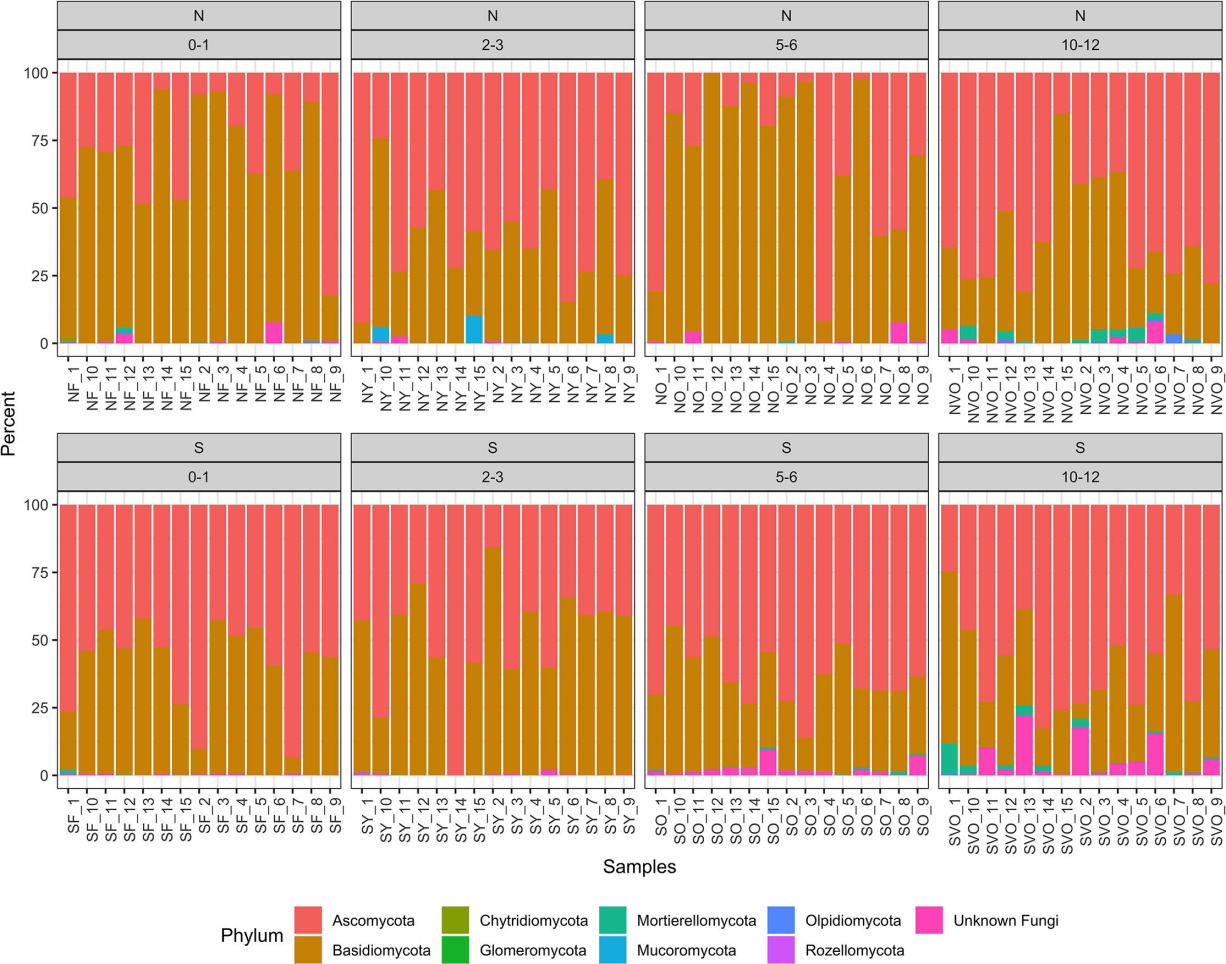
Relative abundance values of phylum hierarchical level of the fungal amplicon sequence variants (ASVs) for every stump sampled at all the locations.

A total of 5,940 ASVs with 6,712,702 reads were detected in the 120 samples from the eight location in Minnesota. The main phyla present were Ascomycota, Basidiomycota, Mucoromycota, Mortierellomycota, and Chytridiomycota were present with Ascomycota (50%) and Basidiomycota (48%) being the most dominant (Figure 1). The taxa identified as *Phlebiopsis gigantea, Resinicium bicolor, Hypochnicium cremicolor, Capronia kleinmondensis, Scytinostroma* sp., *Ciliolarina pinicola, Perenniporia subacida, Nakazawaea ernobii, Xenopolyscytalum pinea, Phialocephala lagerbergii*, and *Sporothrix* sp. were the most dominant at the genus/species level with >100,000 reads (Table 3). Reads assigned to the higher hierarchical level of kingdom, phylum, class, order or family was 1,109,730 of the 6,712,702 total reads or 17%. Thus, 83% of the reads were identified to the genus level and 66% of the genus level reads were identified to species. Additionally, 97,944 or 1% of the total 6,712,702 reads could only be classified to the level of Fungi. The number of unclassified sequences not aligning to a reference sequence increased by aligning to higher taxonomic levels. At the phylum level, the mean percentage of sequences not assigned was 1%. At the class, order, family, genus, and species level the mean percentage of sequences not assigned was 5, 9, 13, 17, and 34% respectively.

**Table 3:** Most abundant taxa identified to the genus or species level for each site for isolations and high-throughput sequencing.

**Taxa**	**No. of isolates**	**Percent of isolates**	**Taxa**	**No. of reads**	**Percent of reads**	**Presence in stumps**
**Native—harvested 0–1 years before sampling**						
*Phlebiopsis gigantea*	13	39%	*Phlebiopsis gigantea*	472,947	58%	15/15
*Bjerkandera adusta*	3	9%	*Diplodia* sp.	35,985	4%	5/15
*Alternaria alternata*	2	6%	*Peniophora pseudopini*	31,969	4%	4/15
**Non-native—harvested 0–1 years before sampling**						
*Phlebiopsis gigantea*	9	24%	*Phlebiopsis gigantea*	257,881	27%	15/15
*Scytalidium album*	3	8%	*Capronia kleinmondensis*	63,596	7%	15/15
*Diplodia sapinea*	3	8%	*Sugiyamaella paludigena*	42,527	4%	2/15
**Native—harvested 2–3 years before sampling**						
*Phlebiopsis gigantea*	14	30%	*Phlebiopsis gigantea*	184,121	20%	15/15
*Leptographium terebrantis*	8	17%	*Nakazawaea ernobii*	134,259	15%	12/15
*Trichoderma deliquescens*	4	9%	*Resinicium bicolor*	127,110	14%	9/15
**Non-native—harvested 2–3 years before sampling**						
*Metapochonia bulbillosa*	3	9%	*Hypochnicium cremicolor*	214,509	24%	11/15
*Ophiostomatales* sp.	3	9%	*Peniophorella pubera*	72,364	8%	7/15
*Trichoderma harzianum*	3	9%	*Xenopolyscytalum pinea*	64,944	7%	14/15
**Native—harvested 5–6 years before sampling**						
*Ophiostomatales* sp.	4	11%	*Resinicium bicolor*	255,972	27%	8/15
*Hormonema macrosporum*	3	8%	*Scytinostroma* sp.	123,296	13%	9/15
*Metapochonia bulbillosa*	3	8%	*Perenniporia subacida*	107,470	11%	6/15
**Non-native—harvested 5–6 years before sampling**						
*Metapochonia bulbillosa*	6	16%	*Boidinia furfuracea*	63,133	8%	15/15
*Irpex lacteus*	4	11%	*Ciliolarina* sp.	41,147	5%	13/15
*Ophiostomatales* sp.	4	11%	*Phialocephala lagerbergii*	37,911	5%	13/15
**Native—harvested 10–12 years before sampling**						
*Umbelopsis isabellina*	6	19%	*Scytinostroma* sp.	61,561	8%	6/15
*Pholiota spumosa*	3	10%	*Ciliolarina pinicola*	46,635	6%	14/15
*Scytalidium album*	3	10%	*Perenniporia subacida*	38,827	5%	4/15
**Non-native—harvested 10–12 years before sampling**						
*Metapochonia bulbillosa*	8	24%	*Ciliolarina pinicola*	46,522	7%	11/15
*Mariannaea elegans*	5	15%	*Xenopolyscytalum pinea*	34,318	5%	15/15
*Alternaria alternata*	2	6%	*Ciliolarina* sp.	23,516	3%	8/15

*Percent of isolates and reads is representative of the particular site and not the overall total. The presence in stumps indicates the recovery rate of ASVs from stumps*.

For sites harvested 0–1 years before sampling, *P. gigantea* was the most abundant identified to the species level at both the northern and southern location present in 30 of 30 samples with 14% of the total reads (Table 3). However, *P. gigantea* was more abundant at the northern location with 472,947 reads (58%) compared to 257,881 reads (27%) at the southern location. Other taxa were present at a much lower level with *Diplodia* sp. as the second most abundant in the northern location representing 4% of the reads and in 5 of 15 stumps. *Capronia kleinmondensis* was the second most abundant in the southern location representing 7% of the reads, but present in 15 of 15 stumps at low levels.

At sites harvested 2–3 years before sampling, *P. gigantea* again was the most abundant at the northern location with 184,121 reads (20%) present in all stumps, but only 5,317 reads (0.61%) and present in just four stumps at the southern location. The yeast *Nakazawaea ernobii* (15%) and the Basidiomycota *Resinicium bicolor* (14%) were also fairly present in northern stumps. The Basidiomycota *Hypochnicium cremicolor* was the most abundant in southern stumps with 201,394 reads (24%) present in 11 of 15 stumps.

*Resinicium bicolor* was the most abundant in northern stumps harvested 5–6 years before sampling with 255,972 reads (27%) present in 8 of 15 stumps. Two other Basidiomycota, *Scytinostroma* sp. (13%), and *Perenniporia subacida* (11%) were comparable in their abundance in northern stumps. A fair number of reads were unclassified at the genus level in southern stumps with 47% of the total reads. The most abundant taxa in southern stumps at the genus/species level were the Basidiomycota *Boidinia furfuracea* (8%), and the Ascomycota *Ciliolarina* sp. (5%).

Both the northern and southern sites had a moderate number of unclassified reads at the genus level in sites harvested 10–12 years before sampling with 22 and 44% respectively. In northern stumps, *Scytinostroma* sp. (8%) and *Ciliolarina pinicola*. (6%) were the most abundant. *Ciliolarina pinicola* (7%) and *Xenopolyscytalum pinea* (5%) were the most abundant in southern stumps at the genus/species level.

### Diversity of Amplicon Sequence Variants

**Figure 2:**
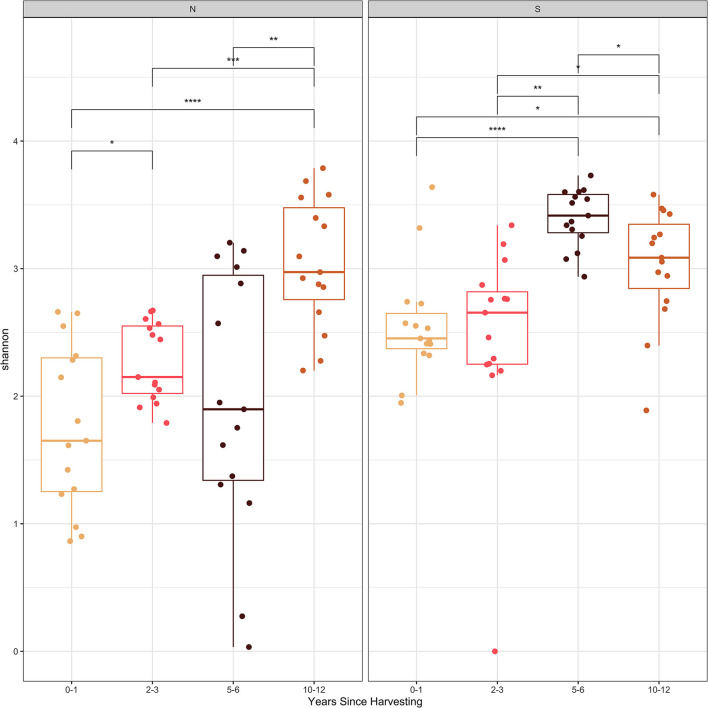
Shannon diversity index of the fungal amplicon sequence variants (ASVs) for every stump sampled at northern (N) and southern (S) locations at different years since harvesting. P-value significance levels indicated as no symbol > 0.05, * ≤ 0.05, ** ≤ 0.01, *** ≤ 0.001, and **** ≤ 0.0001.

**Figure 3:**
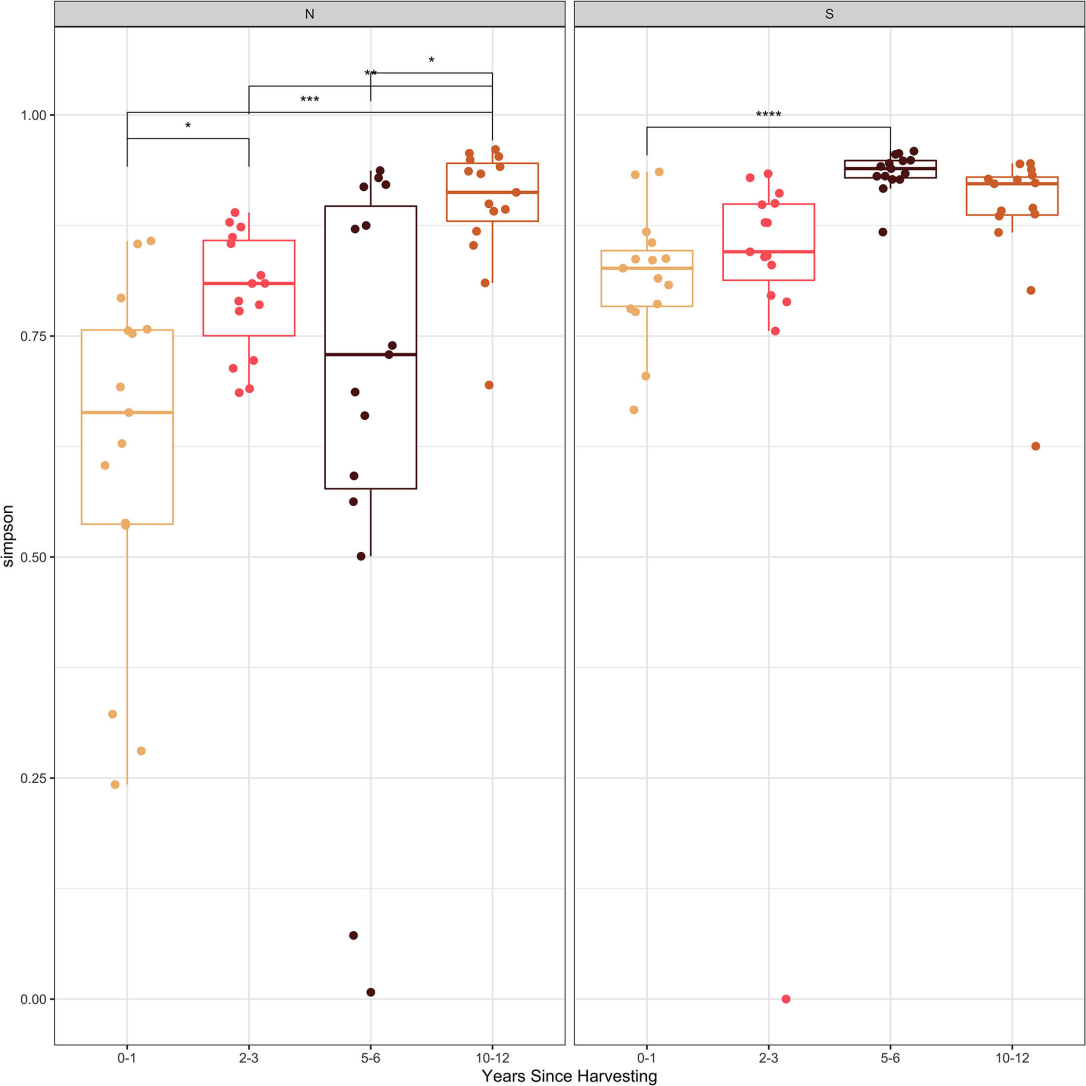
Simpson diversity index of the fungal amplicon sequence variants (ASVs) for every stump sampled at northern (N) and southern (S) locations at different years since harvesting. P-value significance levels indicated as no symbol > 0.05, * ≤ 0.05, ** ≤ 0.01, *** ≤ 0.001, and **** ≤ 0.0001.

The Shannon and Simpson diversity indices (Figures 2, 3) were slightly higher for stumps at the southern locations compared to the northern sites. The diversity gradually increased with years since harvesting for the northern stumps. A similar pattern was observed for the southern stumps, except the stumps harvested 5–6 years ago had the highest diversity. Overall, more variation was observed for diversity at each northern site compared to the southern locations. This is most noticeable for stumps harvested 0–1 and 5–6 years since sampling. All southern stumps had less variation for each sampling period, but some sites were comparable. Additionally, *p*-values were significant when comparing within northern and southern sites.

**Figure 4:**
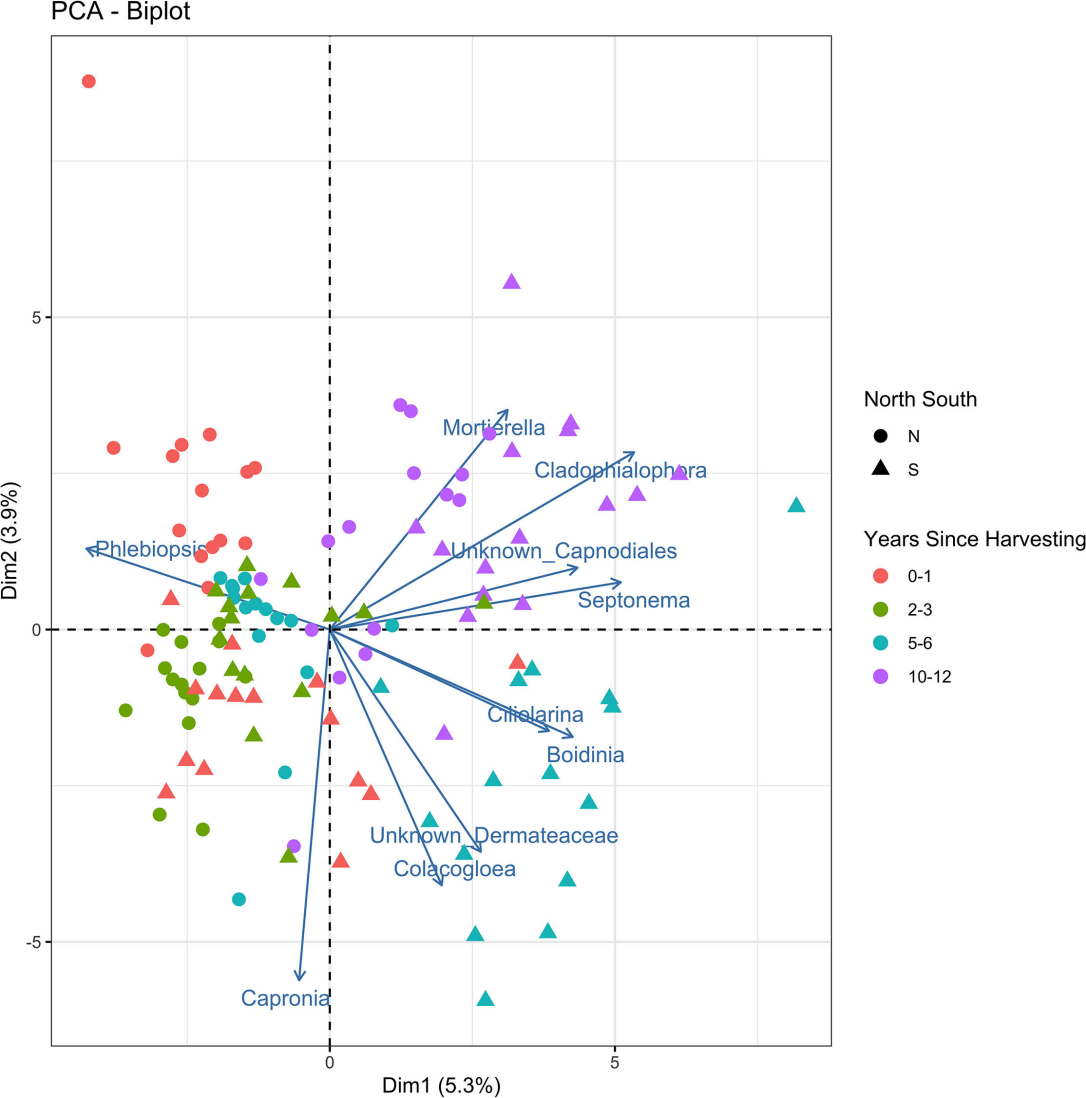
Principal components analysis biplot displaying the beta diversity of the fungal amplicon sequence variants (ASVs) for every stump sampled at northern (N) and southern (S) locations at different years since harvesting.

The principle components analysis (PCA) biplot for northern and southern stumps sampled at different years since harvesting (Figure 4) demonstrated a general correlation of northern stumps from different sites and harvesting times together and southern stumps together. *Phlebiopsis* demonstrates that it is mostly represented in northern stumps as more northern samples are associated with the *Phlebiopsis* vector than the southern stump samples. The PCA biplot also demonstrated a general correlation of stumps based on harvesting year. There is some overlap with stumps recently harvested, 0–1 and 2–3 years ago. However, there is a clear grouping of most stumps harvested 5–6 years ago and the majority of stumps harvested 10–12 years ago.

## Discussion

### Comparison of Fungi Isolated and ASVs Identified

There were some overall similarities between the fungi that were isolated by culturing from stumps and the ASVs identified from stumps. However, some major differences were also evident (Table 3). Primarily the focus is on fungi identified to the genus/species level. One similarity was the abundance of *P. gigantea* in both culturing and Illumina sequencing. Overall, *P. gigantea* was present in 13% of isolations and 14% of reads. Additionally, it was identified in both 19% of isolations and reads in the native range of red pine. In the non-native range of red pine, it was identified in 7% of isolations and 8% of reads. Thus, very similar results for isolations and reads. The similarities were also evident with different sampling times since harvest and by the location of north (native sites) and south (non-native sites). *Phlebiopsis gigantea* was most abundant on stumps that were recently cut (harvested 0–1 and 2–3 years before sampling). Overall, it was found in 25% of isolations and 26% of reads for these stumps. It was also found in 34% of isolations and 38% of reads in the native range of red pine and 14% in both the isolations and reads in the non-native range. Furthermore, it was isolated from 90% of all these stumps in the native range and 33% in the non-native range. Only one isolate and a small number of reads were obtained for *P. gigantea* at sites harvested more than 5 years since sampling. The presence of *P. gigantea* being dramatically reduced in stumps harvested greater than five years before sampling was also consistent with other studies (Vainio et al., 2001; Vasiliauskas et al., 2005).

The northern sites that were recently harvested within 3 years of sampling had a higher presence of *P. gigantea* via culturing and Illumina sequencing than the southern sites. *Phlebiopsis gigantea* was the most abundant fungus isolated and sequenced from the southern site harvested 0–1 years before sampling, but at a lower level. The southern site harvested 2–3 years before sampling had a very low amount of *P. gigantea* reads and just one isolate. A variety of fungi were isolated, but the most abundant fungus sequenced at this site was *Hypochnicium cremicolor*, representing 24% of the total reads. The genus *Hypochnicium* is composed of corticoid, wood-inhabiting, resupinate fungi (Telleria et al., 2010). This fungus could be an early colonizer and may help to prevent *Heterobasidion irregulare* from colonizing stumps. To date, however, no antagonistic studies and biocontrol potential have been investigated with *H. cremicolor* and *H. irregulare*.

A possible reason for sites in southeastern Minnesota having a smaller presence of *P. gigantea* is that these sites are predominately former agriculture land or are isolated red pine stands. Red pine that was planted on sites that were not forested for many years, might not have the population of *P. gigantea* that exists in northern Minnesota, where sites have been forested for many years. Most red pine in northern Minnesota are also not found in localized, isolated stands, but occur more continuously and are less segmented.

The growth of *P. gigantea* mycelium might also be promoted in wood that is growing on forest soils. A previous study found that Scots pine growing in previously fertilized (post-agricultural) soils had different physical and chemical characteristics in wood cell walls and had a lower wood density compared to pines growing in forest soils (Tomczak and Jelonek, 2013). These differences may affect the growth and sporulation of *P. gigantea*. It's also been demonstrated that *P. gigantea* can have rapid growth in Scots pine wood with high specific gravity (Sierota, 1997). Thus, faster growth of *P. gigantea* mycelium could be promoted from wood with a higher density found on forest soils. HRD also has been shown to be more damaging on former agricultural land and pastures than on forest soils (Stenlid and Redfern, 1998). This could be due to a lower amount of antagonistic fungi in these soils, particularly alkaline soils (Rishbeth, 1949, 1950, 1951; Gibbs, 1967). Former agricultural sites also have more subsoil compaction, which can increase the number of root contacts and promote the vegetative spread of *H. irregulare* from tree to tree (Ankudinov, 1950; Day, 1952; Dimitri, 1969).

The large presence of *P. gigantea* in Minnesota identified in this study might be serving as a naturally occurring example of a biological control agent, especially in northern Minnesota. *Phlebiopsis gigantea* colonized cut stumps early and likely is preventing *H. irregulare* from colonizing. The natural deposition of airborne *P. gigantea* spores restricting airborne infections by *Heterobasidion* has been hypothesized previously in Europe (Rishbeth, 1952, 1963; Meredith, 1960; Greig, 1976; Negrutsky, 1986; Holdenrieder and Greig, 1998). However, recent research suggests that natural infection of *P. gigantea* on stumps of *Picea abiea* and *Pinus sylvestris* is not able to effectively restrict infection from *Heterobasidion* spp. (Gaitnieks et al., 2020). This study does involve different hosts and different species of *Heterobasidion*, which could differ compared to the interaction of the hosts and *H. irregulare* in Minnesota. Additionally, in areas where *H. irregulare* is not established and the spore load is low, *P. gigantea* could be acting as an effective biological control agent compared to where *H. irregulare* is established and where spore deposition would be higher.

*Heterobasidion irregulare* was not detected in this study through culturing and Illumina sequencing. As stated previously, *H. irregulare* can be difficult to isolate, but using high-throughput sequencing technology provided an opportunity to use a new method of detection. Previous work has detected spores of *H. irregulare* in Minnesota, but at an amount that most likely would not result in an infection of cut stumps (Otto et al., 2021). This study helps provide further evidence that *H. irregulare* is not widely established at the sites studied in Minnesota. With many *H. irregulare* infection sites existing in Wisconsin and several of these located in counties that border Minnesota, there is a continuous threat from this pathogen. Spore surveys are underway in Minnesota to monitor for this pathogen (Otto et al., 2021).

Other abundant taxa identified from northern sites harvested 2–3 years before sampling included *N. ernobii* and *R. bicolor*. These represented 15% and 14% of the reads and were present in 12 and nine stumps respectively for this site. Additionally, no isolates were obtained of these species during the culturing studies, but one isolate was obtained of *N. holstii* at this site. Additionally, one isolate of *N*. sp was isolated at the northern site harvested 0–1 years before sampling. *N. ernobii* is an ascomycetous yeast that has previously been isolated from decayed wood (Jiménez et al., 1991). Yeast can be common in decayed wood and can be present in both the initial and advanced stages of wood decay (Blanchette and Shaw, 1978). *Resinicium bicolor* is a widespread primary wood degrader that is reported to be common on recently cut wood, but it can also colonize after *P. gigantea* (Vasiliauskas et al., 2005). Another factor that appears to help *R. bicolor* persist, is its production of rhizomorphs. It's been noted that species that producing hyphal aggregates, such as rhizomorphs, have a high general ability to replace other fungi growing in substrates (Stenlid et al., 2008). Rhizomorphs can increase the ability to find resources and allow for import of energy and nutrients thus increasing inoculum potential and the chance of out-competing other mycelia. It's also been noted that *R. bicolor* might be classified as a saprotroph that can establish latently and cause limited decay after wounding (Vasaitis, 2013). However, it's been noted that fungi latently present in the sapwood may not serve a key role as pioneer fungi in conifers. but in angiosperms where fungi are latently present in sapwood they appear to serve a key role as pioneer colonists (Boddy, 2001). The interaction of *R. bicolor* with *H. irregulare* has also been studied and has been shown to be a natural antagonist similar to *P. gigantea* (Holdenrieder and Greig, 1998). It's role as a possible native biological control agent is due to its strong competitive ability, its persistence in stumps and low pathogenicity. Overall, *R. bicolor* was vastly more abundant at northern native growing sites than southern non-native sites.

An abundant fungus isolated at the northern site harvested 2–3 years before sampling was *Leptographium terebrantis*. This fungus was isolated in 31% of the Ascomycota isolates for this site. However, there was a small number of *Leptographium* reads detected in just four stumps total including two at this location. The genus *Leptographium* along with other ophiostomatoid genera cause a discoloration of the sapwood known as blue-stain. Additionally, *L. terebrantis* is a fungus that is carried by the red turpentine beetle that can feed on the lower stem and roots of red pine resulting in red pine pocket mortality (Wisconsin, 2021). This fungus could also be causing unexplained pocket mortality that is not the result of Heterobasidion or Armillaria root rot in Minnesota.

At the genus level, there was a moderate number of unclassified reads for the southern sites harvested 0–1 and 2–3 years before sampling. These represented 16% and 13% of the total reads respectively, at the genus level when compared to both northern sites where 6% represented the unclassified reads at the genus level. Other taxa abundant at these southern sites were *Capronia kleinmondensis* at the site harvested 0–1 years before sampling and *Peniophorella pubera* and *Xenopolyscytalum pinea* at the site harvested 2–3 years before sampling. The majority of the genus *Capronia* is known to occur on decomposing wood or bark (Untereiner, 2000). Species in the *Peniophorella* are wood-inhabiting, resupinate Basidiomycota reported from all forested continents of the world (Hallenberg et al., 2007). The *Xenopolyscytalum* genus belongs to the Helotiales order and are known to be saprotrophic on decaying wood (Zhao et al., 2020).

The northern and southern site harvested 5–6 years before sampling differed overall with isolations and ASVs. The most abundant taxa identified at the northern site was *R. bicolor*, which was also present in 8 of 15 stumps. This species was discussed earlier regarding its ability to readily colonize stumps and its antagonistic properties toward *H. annosum* s.l. Other abundant ASVs were identified as *Scytinostroma* sp. and *Perenniporia subacida. Scytinostroma* sp. was in 9 of 15 stumps and is contributing to the deterioration of the stumps, but *Scytinostroma galactinum* has also been regarded as a facultative saprobe because of its potential role as a pathogen (Lentz and Burdsall, 1973). *Scytinostroma* sp. was also the most abundant Basidiomycota isolated at this site, but just represented 18% of the Basidiomycota isolates. *Perenniporia subacida*, present in 6 of 15 stumps, is a white rot fungus and is considered a weak pathogen of conifers (Sinclair and Lyon, 2005).

The southern site harvested 5–6 years before sampling also had a moderate number of unclassified reads at the genus level with 23%. The most abundant taxa identified was *Boidinia furfuracea*, present in 15 of 15 stumps. The genus *Boidinia* is a thin, inconspicuous corticoid fungus that mainly lives as a saprophyte on different kinds of dead wood (Eriksson and Ryvarden, 1975). Other abundant taxa were *Ciliolarina* sp. and *Ciliolarina pinicola*. In a study examining fungal communities in dead wood of Scots pine, *Cilolarina* spp. was found commonly on wood in their second decay class, which was 5–20 years after felling (Behnke-Borowczyk et al., 2021). This aligns with the red pine wood in this study that was felled 6 years before sampling. *Phialocephala lagerbergii* was also abundant and present in 13 of 15 stumps. This genus is known to be commonly found in roots and decayed wood in forest ecosystems across north temperate regions and also displays remarkable plasticity (Addy et al., 2000; Grünig et al., 2009). The most common Basidiomycota isolated at the southern site was *Irpex lacteus*. It was also the most abundant fungus isolated from dead and dying trees and stumps in previous work surveying for *H. irregulare* (Otto et al., 2021). This confirms the previous study indicating it is more common on pine hosts than previously thought. No ASVs were identified as *I. lacteus*, but *I. hydnoides* was identified in low amounts at the northern and southern sites harvested 0–1 years before sampling. *Metapochonia bulbillosa* was also commonly isolated from the southern site. This fungus was commonly isolated in a previous study examining the abundance and diversity of fungi in Scots pine deadwood in Poland (Kwaśna et al., 2017). *Metapochonia* sp. and the similar genus *Pochonia* sp. were only detected in one stump each at the southern and northern site harvested 0–1 years before sampling with a very low number of reads. However, the species *M. goniodes* was more prevalent, particularly at the southern sites harvested 5–6 and 10–12 years before sampling being identified in 25 of 30 stumps, but with a low number of reads.

Both the northern and southern site harvested 10–12 years before sampling had a high number of unclassified reads with 22 and 44% at the genus level, respectively. Other abundant ASVs in the northern site were identified as *Scytinostroma* sp., *Ciliolarina pinicola*, and *P. subacida*. Both *Scytinostroma* sp. and *P. subacida* were also abundant at the northern site harvested 5–6 years before sampling. *Resinicium bicolor*, which was abundant at the northern site harvested 2–3 and 5–6 years before sampling was not abundant. This helps demonstrate the displacement of *R. bicolor* and the transition to other wood decay species. The most abundant species isolated was *Scytalidium album* at the northern site harvested 10–12 years before sampling. This species has been shown to prevent decay from white and brown rot fungi *in vitro* (Ricard and Bollen, 1968; Cease et al., 1989; Highley, 1990, 1994). Additionally, the genus has demonstrated antagonistic activity *in vitro* against *H. annosum* s.l. (Klingström and Beyer, 1965). The most abundant ASVs at the southern site were identified as *Ciliolarina pinicola* and *Xenopolyscytalum pinea*. Species of *Ciliolarina* were also abundant at the southern site harvested 5–6 years ago and the northern sites harvested 10–12 years before samplings. As mentioned previously, this genus was common on dead Scots pine wood felled 5–20 years ago (Behnke-Borowczyk et al., 2021), which aligns with this set of samples harvested 10–12 years ago. The species *X. pinea* has been found on needles of *Pinus* previously and its role or interactions during wood degradation is uncertain (Crous and Groenewald, 2010). The most commonly isolated species from the site harvested 10–12 years before sampling included *Mariannaea elegans* and *Metapochonia bulbillosa. M. elegans* is known to grow on decaying coniferous bark, wood or forest soil and has been isolated from Scots pine previously (Kwaśna et al., 2017). Additionally, in our study both *M. bulbillosa* and *M. elegans* were more commonly isolated from wood that was felled 5–6 and 10–12 years before sampling, which is consistent with Kwaśna et al. (2017) as both of these species were more abundant at sites harvested 5–20 years before sampling.

The differences between results from culturing and ASVs could be from the methods used for culturing that are known to favor rapidly growing fungi, saprotrophs, and other opportunistic fungi (Bonito et al., 2016). This can lead to a good proportion of isolates being those that will quickly grow on media or outcompete other fungi. Thus, the fungi isolated would be expected to be less than the diversity of ASVs obtained through high-throughput sequencing. However, there were fungal isolates that were not present in the sequencing data. This could be due to not currently being in the SILVA sequence datasets, but present in the sequence database for the National Center for Biotechnology Information. Overall, there were some general similarities, but the difference between the species isolated and ASVs identified were substantive at times. The use of high-throughput sequencing allows for a richer way to identify fungi present in a given substrate, but users still need to be aware of methodological biases and bioinformatics challenges (Lindahl et al., 2013). One particular challenge is that it is unclear whether the ITS1 or ITS2 region of fungal ribosomal DNA (rDNA) is best suited for metabarcoding complex fungal communities (Frau et al., 2019). It was thought that ITS1 was more variable and would allow better distinction among species than ITS2 (Nilsson et al., 2008). However, the opposite has also been shown for ITS2 indicating it would be better suited (Bazzicalupo et al., 2013; Tedersoo et al., 2015). The use of ITS1 in this study could be a reason a very small amount of reads of *Leptographium* were obtained even though it was commonly isolated as previously mentioned. Performing additional sequencing with ITS2 might further elucidate the fungal community present in these stumps. Unclassified reads and unknown species might then be potenitially revelaed with ITS2. Overall, both culturing and high-throughput sequencing are useful and should continue to be used and preferably in combination to assess for fungal diversity.

Abundant taxa identified, such as *P. gigantea* were present in most of the stumps sampled at a given site. However, some abundant taxa, such as *R. bicolor*, were not present in all the stumps sampled and sometimes found in just over half of the stumps. These dominant fungal species, such as *R. bicolor*, could be unique on individual stumps and help reflect the stochastic nature of fungal colonization (Van Der Wal et al., 2015). This stochastic nature could be due to fungal colonization methods such as colonization by wind or insect dispersed spores, mycelial fragments (Persson et al., 2011) or endophytic fungi already present in living trees (Parfitt et al., 2010).

The community of fungi that develops in substrates is not a simple ordered sequence, but an ever-changing mosaic that can be complex (Boddy and Hiscox, 2017). This study did not examine the same site over time, but sampled sites of different ages, which adds to the complexity. Conducting a study using the same location sampled over a 10 year period would provide additional information.

### ASV Diversity

Examining the Shannon and Simpson diversity indices at the northern and southern sites reveals a slightly lower diversity at northern sites sampled 0–1 and 2–3 years after harvesting. Both the Shannon and Simpson diversity indices (Figures 2, 3) showed similar results. Southern sites showed a higher diversity, most likely due to not having the abundant presence of *P. gigantea, R. bicolor*, and *Scytinostroma* sp. in large amounts compared to the northern sites.

A lower diversity at native red pine sites in northern locations could be due to the greater amount of *P. gigantea* present and the ability of this fungus to prevent a higher diversity of pioneer species to attack fresh stumps. If a higher diversity of pioneer species were allowed to colonize fresh stumps, then more microhabitats could be created leading to a higher diversity of successive species (Vasiliauskas et al., 2005).

Generally, species richness and diversity will not change significantly with fungal succession as it is a complex and unpredictable process (Jumpponen et al., 2012; Martínez-García et al., 2015). This is mostly true for all sites sampled in our study, but there is slight trend of increase for both Shannon and Simpson diversity indices with time since harvesting. This is more evident for the northern sites. This is also evident, specifically with sites harvested over 5 years ago with the majority of the most abundant taxa not reaching 10% of the reads (Table 3). Finally, the significant *p*-values help indicate the difference in species composition within the northern and southern sites. This further emphasizes the change in species composition with years since harvesting.

As mentioned previously, the majority of northern and southern stumps appear to be separate and generally grouped together in the PCA biplot (Figure 4). The vector of *Phlebiopsis* demonstrates that it is mostly negatively correlated with other fungi present in stumps due to the large angles between vectors with other fungi. As mentioned previously, recently harvested stumps where *P. gigantea* is dominate leads to less diversity of other fungi and thus, no to negative correlation with other fungal vectors. There also appears to be the most positive correlation between fungi in stumps harvested 10–12 years before sampling. This is most likely due to no fungal species being dominate in these stumps.

Evaluating the decay stage of stumps (Table 1) showed that sites harvested 0–1 years before sampling had stumps primarily in decay class I. Sites harvested 2–3 years before sampling had stumps in decay class I–II, sites harvested 5–6 years before sampling were primarily III, and sites harvested 10–12 years ago were in decay class IV. Previous studies have observed an increase in fungal species richness with wood decay (Rajala et al., 2011; Van Der Wal et al., 2015). This is similar to the study reported here where stumps harvested 10–12 years before sampling had a higher alpha and beta diversity.

## Conclusion

No previous study has examined the fungal composition of red pine stumps grown on native and non-native locations with a combination of isolations and high-throughput sequencing. A chronosequence was also established by examining stumps harvested at different times before sampling. This study demonstrated the abundance of *P. gigantea* on recently harvested stumps with a higher amount at northern sites where red pine is in its native range. This fungus could be acting as a native biological control agent helping prevent *H. irregulare* from becoming established in Minnesota. Additionally, no isolates or ASVs were obtained for *H. irregulare* during the duration of this study indicating that this pathogen has not become well established in Minnesota. Other fungi, such as *C. kleinmondensis, R. bicolor*, and *H. cremicolor* were also found to be pioneer species colonizing pine stumps soon after cutting, but at a much lower level than *P. gigantea*. In addition, a diverse group of wood decay fungi were found and differences observed between sites where red pine is growing in its native range as compared to non-native locations.

## Data Availability Statement

The Illumina MiSeq data presented in the study can be found in the NCBI Sequence Read Archive with the following BioProject accession number: PRJNA776966.

## Author Contributions

EO and RB: designed the experiments. EO, BH, and RB: performed the field work. EO and BH: performed the isolations and molecular methods. EO and TG: analyzed the data. EO: wrote the original draft. EO, RB, BH, and TG: wrote, revised, and edited the paper. RB: acquired funding for the project. All authors contributed to the article and approved the submitted version.

## Funding

This research was funded by the Minnesota Environment and Natural Resources Trust Fund (ENRTF) and USDA Hatch Project MIN-0022081 and MIN-0022089.

## Conflict of Interest

The authors declare that the research was conducted in the absence of any commercial or financial relationships that could be construed as a potential conflict of interest.

## Publisher's Note

All claims expressed in this article are solely those of the authors and do not necessarily represent those of their affiliated organizations, or those of the publisher, the editors and the reviewers. Any product that may be evaluated in this article, or claim that may be made by its manufacturer, is not guaranteed or endorsed by the publisher.

## References

[B1] Addy H. D. Hambleton S. Currah R. S. (2000). Distribution and molecular characterization of the root endophyte *Phialocephala fortinii* along an environmental gradient in the boreal forest of Alberta. Mycol. Res. 104, 1213–1221. 10.1017/S095375620000289630886898

[B2] Ankudinov A. M. (1950). Death of pine plantations on former arable land. Lesnoe Khozyaistvo 9, 46–49.

[B3] Bazzicalupo A. L. Bálint M. Schmitt I. (2013). Comparison of ITS1 and ITS2 rDNA in 454 sequencing of hyperdiverse fungal communities. Fungal Ecol. 6, 102–109. doi.: 10.1016/j.funeco.2012.09.003

[B4] Behnke-Borowczyk J. Kwasna H. Kartawik N. Sijka B. Belka M. Lakomy P. (2021). Effect of management on fungal communities in dead wood of Scots pine. For. Ecol. Manag. 479:528. 10.1016/j.foreco.2020.118528

[B5] Blanchette R. A. (1995). Degradation of the lignocellulose complex in wood. Can. J. Bot. 73, 999–1010. 10.1139/b95-350

[B6] Blanchette R. A. Held B. W. Hellmann L. Millman L. Büntgen U. (2016). Arctic driftwood reveals unexpectedly rich fungal diversity. Fungal Ecol. 23, 28–65. 10.1016/j.funeco.2016.06.001

[B7] Blanchette R. A. Held B. W. Jurgens J. Stear A. Dupont C. (2021). Fungi attacking historic wood of fort conger and the peary huts in the high arctic. PLOS ONE 16:e0246049. 10.1371/journal.pone.0246049 33497418 PMC7837483

[B8] Blanchette R. A. Held B. W. Mollov D. Blake J. D'Amato A. W. (2015). First report of *Heterobasidion irregulare* causing root rot and mortality of red pines in Minnesota. Plant Dis. 99, 1038–1038. 10.1094/PDIS-11-14-1232-PDN30812563

[B9] Blanchette R. A. Shaw C. G. (1978). Associations among bacteria, yeasts, and basidiomycetes during wood decay. Phytopathology 68, 631–637. 10.1094/Phyto-68-63130812563

[B10] Boddy L. (2001). Fungal community ecology and wood decomposition processes in angiosperms : from standing tree to complete decay of coarse woody debris. Ecol. Bull. 49, 43–56. 10.3389/fmicb.2016.00231 26973611 PMC4771932

[B11] Boddy L. Heilmann-Clausen J. (2008). Chapter 12 Basidiomycete community development in temperate angiosperm wood. Br. Mycol. Soc. Sympo. Series 28, 211–237. 10.1016/S0275-0287(08)80014-8

[B12] Boddy L. Hiscox J. (2017). fungal ecology: principles and mechanisms of colonization and competition by saprotrophic fungi. Fung. King. 13, 293–308. 10.1128/9781555819583.ch13 28087930

[B13] Bonito G. Hameed K. Ventura R. Krishnan J. Schadt C. W. Vilgalys R. (2016). Isolating a functionally relevant guild of fungi from the root microbiome of *Populus*. Fungal Ecol. 22, 35–42. 10.1016/j.funeco.2016.04.007

[B14] Callahan B. J. McMurdie P. J. Rosen M. J. Han A. W. Johnson A. J. A. Holmes S. P. (2016). DADA2: High-resolution sample inference from Illumina amplicon data. Nat. Methods 13, 581–583. 10.1038/nmeth.3869 27214047 PMC4927377

[B15] Cease K. R. Blanchette R. A. Highley T. L. (1989). Interactions between *Scytalidium* species and brown- or white-rot basidiomycetes in birch wood. Wood Sci. Technol. 23, 151–161.

[B16] Cooke R. C. Rayner A. D. M. (1984). Ecology of Saprotrophic Fungi. London: Wiley.

[B17] Crous P. W. Groenewald J. Z. (2010). *Xenopolyscytalum pinea*, gen. and sp. nov. Fungal planet 55. Persoonia 25, 130–131.

[B18] Day W. R. (1952). Root disease of conifers in relation to soil conditions, in (A) Development of butt-rot in relation to soil depth. (B) The dying of Sitka spruce. Imperial Forestry Institute Report, New York, NY: Oxford.

[B19] Dimitri L. (1969). Untersuchungen uber die unteridischen Eintrittspforten der wichtigsten Rotfauleerreger bei der Fichte (Picea abies Karst.) Forstwissenschaftliches Centralblatt 88, 281–308. 10.1007/BF02741785

[B20] Dumas M. T. Laflamme G. (2013). Efficacy of two *Phlebiopsis gigantea* formulations in preventing *Heterobasidion irregulare* colonization of red pine stumps in eastern Canada. Phytoprotection 93, 25–31. 10.7202/1018887ar

[B21] Eriksson J. Ryvarden L. (1975). The Corticiacae of North Europe. Oslo: Fungiflora, 549–884.

[B22] Eriksson K.-E. L. Blanchette R. A. Ander P. (1990). Morphological Aspects of Wood Degradation by Fungi and Bacteria, in Microbial and Enzymatic Degradation of Wood and Wood Componentts. Springer Series in Wood Science. Berlin, Heidelberg: Springer, 1–87.

[B23] Frau A. Kenny J. G. Lenzi L. Campbell B. J. Ijaz U. Z. Duckworth C. A.. (2019). DNA extraction and amplicon production strategies deeply inf luence the outcome of gut mycobiome studies. Sci. Rep. 9:1. 10.1038/s41598-019-44974-x 31249384 PMC6597572

[B24] Gaitnieks T. Zaluma A. Kenigsvalde K. Bruna L. Klavina D. Burnevica N.. (2020). Natural infection and colonization of pre-commercially cut stumps of *Picea abies* and *Pinus sylvestris* by *Heterobasidion* rot and its biocontrol fungus *Phlebiopsis gigantea*. Biol. Control. 143:104208. 10.1016/j.biocontrol.2020.104208

[B25] Garbelotto M. Gonthier P. (2013). Biology, Epidemiology, and Control of *Heterobasidion* Species Worldwide. Annu. Rev. of Phytopathol. 51, 39–59. 10.1146/annurev-phyto-082712-102225 23642002

[B26] Gardes M. Bruns T. D. (1993). ITS primers with enhanced specificity for basidiomycetes - application to the identification of mycorrhizae and rusts. Mol. Ecol. 2, 113–118. 10.1111/j.1365-294X.1993.tb00005.x 8180733

[B27] Gibbs J. N. (1967). A study of the epiphytic growth habit of *Fomes annosus*. Ann. Bot. 31, 755–774. 10.1093/oxfordjournals.aob.a084180

[B28] Gilmore D. W. Palik B. J. (2005). A Revised Managers Handbook for Red Pine in the North Central Region. General Technical Report NC-264. Growth (Lakeland).

[B29] GitHub (2016). karthik/wesanderson. Available online at: https://github.com/karthik/wesanderson

[B30] Greig B. J. W. (1976). Biological control of *Fomes annosus* by *Peniophora gigantea*. Eur. J. Plant Pathol. 6, 286–290. 10.1111/j.1439-0329.1976.tb00508.x

[B31] Grünig C. R. Queloz V. Du,ò A. Sieber T. N. (2009). Phylogeny of Phaeomollisia piceae gen. sp. nov.: a dark, septate, conifer-needle endophyte and its relationships to Phialocephala and Acephala. Mycol. Res. 113, 207–221. 10.1016/j.mycres.2008.10.005 19015028

[B32] Hallenberg N. Nilsson R. H. Antonelli A. Wu S. H. Maekawa N. Norden N. (2007). The *Peniophorella praetermissa* species complex (Basidiomycota). Mycol. Res. 111:1366–1376. 10.1016/j.mycres.2007.10.001 18023990

[B33] Heilmann-Clausen J. (2001). A gradient analysis of communities of macrofungi and slime moulds on decaying beech logs. Mycol. Res. 105, 575–596. 10.1017/S095375620100366530886898

[B34] Highley T. L. (1990). Laboratory studies on antagonism of *Scytalidium lignicola* to wood decay fungi. Mater. Org. 25, 181–192.

[B35] Highley T. L. (1994). Effect of Scytalidium lignicola on decay resistance and strength of wood, in International Research Group on Wood Preservation, Document No. IRG/WP/94-10061.

[B36] Holdenrieder O. Greig B. J. W. (1998). Biological methods of control, in Heterobasidion Annosum: Biology, Ecology, Impact, and Control. CABI: Wallingford, 235–258.

[B37] Holmer L. Stenlid J. (1997). Competitive hierarchies of wood decomposing basidiomycetes in artificial systems based on variable inoculum sizes. Oikos 79:77. 10.2307/3546092

[B38] Hori C. Ishida T. Igarashi K. Samejima M. Suzuki H. Master E.. (2014). Analysis of the *Phlebiopsis gigantea* genome, transcriptome and secretome provides insight into its pioneer colonization strategies of wood. PLoS Genet. 10:e1004759. 10.1371/journal.pgen.1004759 25474575 PMC4256170

[B39] Hottola J. Siitonen J. (2008). Significance of woodland key habitats for polypore diversity and red-listed species in boreal forests. Biodivers. Conserv. 17, 2559–2577. 10.1007/s10531-008-9317-4

[B40] Jiménez M. González A. E. Martínez M. J. Martínez A. T. Dale B. E. (1991). Screening of yeasts isolated from decayed wood for lignocellulose-degrading enzyme activities. Mycol. Res. 95, 1299–1302. 10.1016/S0953-7562(09)80578-9

[B41] Johnson E. A. Miyanishi K. (2021). Disturbance and succession. Plant Disturb. Ecol. 10, 1–15. 10.1016/B978-0-12-818813-2.00001-0

[B42] Jumpponen A. Brown S. P. Trappe J. M. Cázares E. Strömmer R. (2012). Twenty years of research on fungal-plant interactions on Lyman Glacier forefront—lessons learned and questions yet unanswered. Fungal Ecol. 5, 430–442. 10.1016/j.funeco.2012.01.002

[B43] Kaitera J. Henttonen H. M. Müller M. M. (2019). Fungal species associated with butt rot of mature Scots pine and Norway spruce in northern boreal forests of Northern Ostrobothnia and Kainuu in Finland. Eur. J. Plant Pathol. 154, 541–554. 10.1007/s10658-019-01678-2

[B44] Kearse M. Moir R. Wilson A. Stones-Havas S. Cheung M. Sturrock S.. (2012). Geneious Basic: an integrated and extendable desktop software platform for the organization and analysis of sequence data. Bioinformatics 28, 1647–1649. 10.1093/bioinformatics/bts199 22543367 PMC3371832

[B45] Klingström A. Beyer L. (1965). Two new species of *Scytalidium* with antagonistic properties to *Fomes annosus* (Fr.) Cke. Svensk Botanisk Tidskrift 59, 30–36.

[B46] Kubart A. Vasaitis R. Stenlid J. Dahlberg A. (2016). Fungal communities in Norway spruce stumps along a latitudinal gradient in Sweden. Forest Ecol. Manag. 371, 50–58. 10.1016/j.foreco.2015.12.017

[B47] Kubartov,á A. Ottosson E. Dahlberg A. Stenlid J. (2012). Patterns of fungal communities among and within decaying logs, revealed by 454 sequencing. Mol. Ecol. 21, 4514–4532. 10.1111/j.1365-294X.2012.05723.x 22882383

[B48] Kwaśna H. Mazur A. Kuzmiński R. Jaszczak R. Turski M. Behnke-Borowczyk J.. (2017). Abundance and diversity of wood-decay fungi in managed and unmanaged stands in a Scots pine forest in western Poland. Forest Ecol. Manag. 400, 438–446. 10.1016/j.foreco.2017.04.023

[B49] Lentz P. L. Burdsall H. H. (1973). *Scytinostroma galactinum* as a pathogen of woody plants. Mycopathol. Mycol. Appl. 49, 289–305. 10.1007/BF02050723

[B50] Lindahl B. D. Nilsson R. H. Tedersoo L. Abarenkov K. Carlsen T. Kjøller R.. (2013). Fungal community analysis by high-throughput sequencing of amplified markers—a user's guide. New Phytol. 199, 288–299. 10.1111/nph.12243 23534863 PMC3712477

[B51] Martin M. (2011). Cutadapt removes adapter sequences from high-throughput sequencing reads. EMBnet. J. 17:10. 10.14806/ej.17.1.200

[B52] Martínez-García L. B. Richardson S. J. Tylianakis J. M. Peltzer D. A. Dickie I. A. (2015). Host identity is a dominant driver of mycorrhizal fungal community composition during ecosystem development. New Phytol. 205, 1565–1576. 10.1111/nph.13226 25640965

[B53] Mbareche H. Veillette M. Bilodeau G. Duchaine C. (2020). Comparison of the performance of ITS1 and ITS2 as barcodes in amplicon-based sequencing of bioaerosols. PeerJ. 2020:2. 10.7717/peerj.8523 32110484 PMC7032056

[B54] Meredith D. S. (1960). Further observations on fungi inhabiting pine stumps. Ann. Bot., New Series 24, 63–78. 10.1093/oxfordjournals.aob.a083689

[B55] Monard C. Gantner S. Stenlid J. (2013). Utilizing ITS1 and ITS2 to study environmental fungal diversity using pyrosequencing. FEMS Microbiol. Ecol. 84, 165–175. 10.1111/1574-6941.12046 23176677

[B56] Negrutsky S. F. (1986). Kornevaya gybka. [Heterobasidion annosum]. Agropromizdat: Moscow, 1–196. [In Russian].

[B57] Nicolotti G. Gonthier P. (2005). Stump treatment against *Heterobasidion* with *Phlebiopsis gigantea* and some chemicals in *Picea abies* stands in the western Alps. For. Pathol. 35, 365–374. 10.1111/j.1439-0329.2005.00419.x

[B58] Nilsson R. H. Kristiansson E. Ryberg M. Hallenberg N. Larsson K. H. (2008). Intraspecific ITS variability in the Kingdom Fungi as expressed in the international sequence databases and its implications for molecular species identification. Evol. Bioinform. 2008, 193–201. 10.4137/EBO.S653 19204817 PMC2614188

[B59] Oksanen J. Blanchet F. G. Friendly M. Kindt R. Legendre P. Mcglinn D. W.. (2020). Package “vegan” Title Community Ecology Package Version 2. 5–7.

[B60] Oliva J. Messal M. Wendt L. Elfstrand M. (2017). Quantitative interactions between the biocontrol fungus *Phlebiopsis gigantea*, the forest pathogen *Heterobasidion annosum* and the fungal community inhabiting Norway spruce stumps. Forest Ecol. Manag. 402, 253–264. 10.1016/j.foreco.2017.07.046

[B61] Otto E. Held B. Redford S. Blanchette R. A. (2021). Detecting *Heterobasidion irregulare* in Minnesota and Assessment of Indigenous Fungi on Pines. Forests 12:57. 10.3390/f12010057

[B62] Ovaskainen O. Nokso-Koivisto J. Hottola J. Rajala T. Pennanen T. Ali-Kovero H.. (2010). Identifying wood-inhabiting fungi with 454 sequencing—what is the probability that BLAST gives the correct species? Fung. Ecol. 3, 274–283. 10.1016/j.funeco.2010.01.001

[B63] Ovaskainen O. Schigel D. Ali-Kovero H. Auvinen P. Paulin L. Nordén B.. (2013). Combining high-throughput sequencing with fruit body surveys reveals contrasting life-history strategies in fungi. ISME J. 7, 1696–1709. 10.1038/ismej.2013.61 23575372 PMC3749500

[B64] Parfitt D. Hunt J. Dockrell D. Rogers H. J. Boddy L. (2010). Do all trees carry the seeds of their own destruction? PCR reveals numerous wood decay fungi latently present in sapwood of a wide range of angiosperm trees. Fungal Ecol. 3, 338–346. 10.1016/j.funeco.2010.02.001

[B65] Pearce M. H. Malajczuk N. (1990). Stump colonization by *Armillaria luteobubalina* and other wood decay fungi in an age series of cut-over stumps in karri (*Eucalyptus diversicolor*) regrowth forests in south-western Australia. New Phytol. 115, 129–138. 10.1111/j.1469-8137.1990.tb00930.x

[B66] Persson Y. Ihrmark K. Stenlid J. (2011). Do bark beetles facilitate the establishment of rot fungi in Norway spruce? Fungal Ecol. 4, 262–269. 10.1016/j.funeco.2011.01.005

[B67] Pratt J. E. Niemi M. Sierota Z. H. (2000). Comparison of three products based on *Phlebiopsis gigantea* for the control of *Heterobasidion annosum* in Europe. Biocontrol Sci. Technol. 10, 467–477. 10.1080/09583150050115052

[B68] Quast C. Pruesse E. Yilmaz P. Gerken J. Schweer T. Yarza P.. (2013). The SILVA ribosomal RNA gene database project: improved data processing and web-based tools. Nucleic Acids Res. 41:D590. 10.1093/nar/gks1219 23193283 PMC3531112

[B69] R Core Team (2017). R: A Language and Environment for Statistical Computing. Vienna: R Foundation for Statistical Computing.

[B70] Rajala T. Peltoniemi M. Hantula J. Mäkip,ää R. Pennanen T. (2011). RNA reveals a succession of active fungi during the decay of Norway spruce logs. Fungal Ecol. 4, 437–448. 10.1016/j.funeco.2011.05.005

[B71] Rajala T. Peltoniemi M. Pennanen T. Mäkip,ää R. (2010). Relationship between wood-inhabiting fungi determined by molecular analysis (denaturing gradient gel electrophoresis) and quality of decaying logs. Can. J. For. Res. 40, 2384–2397. 10.1139/X10-176

[B72] Rajala T. Peltoniemi M. Pennanen T. Mäkip,ää R. (2012). Fungal community dynamics in relation to substrate quality of decaying Norway spruce (*Picea abies* [L.] Karst.) logs in boreal forests. FEMS Microbiol. Ecol. 81, 494–505. 10.1111/j.1574-6941.2012.01376.x 22458543

[B73] Rayner A. D. M. Boddy L. (1988). Fungal Decomposition of Wood; Its Biology and Ecology. Chichester: Wiley.

[B74] Rayner A. D. M. Webber J. F. (1984). Interspecific mycelial interactions-an overview, in Ecology and Physiology of the Fungal Mycelium. Cambridge: Cambridge University Press.

[B75] Ricard J. L. Bollen W. B. (1968). Inhibition of *Poria carbonica* by *Scytalidium* sp., an imperfect fungus isolated from Douglas-fir poles. Can. J. Bot. 46, 643–647. 10.1139/b68-092

[B76] Rishbeth J. (1949). *Fomes annosus* (FR). on pines in Easy Anglia. Forestry 22, 174–183. 10.1093/oxfordjournals.forestry.a062956

[B77] Rishbeth J. (1950). Observations on the Biology of *Fomes annosus*, with particular reference to East Anglian pine plantations. Ann. Bot 14, 365–383. 10.1093/oxfordjournals.aob.a083252

[B78] Rishbeth J. (1951). Observations on the Biology of *Fomes annosus*, with particular reference to East Anglian pine plantations. Ann. Bot. 15, 221–246. 10.1093/oxfordjournals.aob.a083278

[B79] Rishbeth J. (1952). Control of *Fomes annosus* Fr. Forestry 25, 41–50. 10.1093/oxfordjournals.forestry.a06303930727562

[B80] Rishbeth J. (1959). Dispersal of *Fomes annosus* Fr. and Peniophora gigantea (Fr.) Massee. Trans. Brit. Mycol.Soc. 42, 243–260. 10.1016/S0007-1536(59)80034-6

[B81] Rishbeth J. (1963). Stump protection against *Fomes annosus* III. Inoculation with Peniophora gigantea. Ann. Appl. Biol. 52, 63–77. 10.1111/j.1744-7348.1963.tb03728.x

[B82] Schilling J. S. Kaffenberger J. T. Held B. W. Ortiz R. Blanchette R. A. (2020). Using Wood rot phenotypes to illuminate the “gray” among decomposer fungi. Front. Microbiol. 11, 1288. 10.3389/fmicb.2020.01288 32595628 PMC7303305

[B83] Schilling J. S. Kaffenberger J. T. Liew F. J. Song Z. (2015). Signature wood modifications reveal decomposer community history. PLoS ONE 10:120679. 10.1371/journal.pone.0120679 25811364 PMC4374725

[B84] Sierota Z. H. (1997). Dry weight loss of wood after the inoculation of Scots pine stumps with *Phlebiopsis gigantea*. Euro. J. For. Pathol. 27, 179–185. 10.1111/j.1439-0329.1997.tb00859.x

[B85] Sinclair W. A. Lyon H. H. (2005). Diseases of Trees and Shrubs (2nd ed.). Ithaca, NY: Cornell University Press.

[B86] Stanosz G. R. Smith D. R. Juzwik J. (2016). Seasonal availability of inoculum of the Heterobasidion root disease pathogen in central Wisconsin. Can. J. For. Res. 46, 1076–1080. 10.1139/cjfr-2016-0136

[B87] Stenlid J. Penttil,ä R. Dahlberg A. (2008). Chapter 13 Wood-decay basidiomycetes in boreal forests: distribution and community development. Br. Mycol. Soc. Symposia Series 28, 239–262. 10.1016/S0275-0287(08)80015-X

[B88] Stenlid J. Redfern D. B. (1998). Spread within the tree and stand, in Heterobasidion Annosum: Biology Ecology, Impact, and Control. Wallingford: CABI, 125–141.

[B89] Talbot J. M. Martin F. Kohler A. Henrissat B. Peay K. G. (2015). Functional guild classification predicts the enzymatic role of fungi in litter and soil biogeochemistry. Soil Biol. Biochem. 88, 441–456. 10.1016/j.soilbio.2015.05.006

[B90] Tedersoo L. Anslan S. Bahram M. Põlme S. Riit T. Liiv I.. (2015). Shotgun metagenomes and multiple primer pair-barcode combinations of amplicons reveal biases in metabarcoding analyses of fungi. MycoKeys 10, 1–43. 10.3897/mycokeys.10.4852

[B91] Telleria M. T. Dueñas M. Melo I. Hallenberg N. Martín M. P. (2010). A re-evaluation of *Hypochnicium* (Polyporales) based on morphological and molecular characters. Mycologia 102, 1426–1436. 10.3852/09-242 20943566

[B92] Terhonen E. Sun H. Buée M. Kasanen R. Paulin L. Asiegbu F. O. (2013). Effects of the use of biocontrol agent (*Phlebiopsis gigantea*) on fungal communities on the surface of *Picea abies* stumps. For. Ecol. Manag. 310, 428–433. 10.1016/j.foreco.2013.08.044

[B93] Tomczak A. Jelonek T. (2013). Radial variation in the wood properties of Scots pine (*Pinus sylvestris* L.) grown on former agricultural soil. For. Res. Pap. 74, 171–177. 10.2478/frp-2013-0017

[B94] Untereiner W. A. (2000). *Capronia* and its anamorphs: exploring the value of morphological and molecular characters in the systematics of the Herpotrichiellaceae. Stud. Mycol. 45:141–148.

[B95] Vainio E. J. Lipponen K. Hantula J. (2001). Persistence of a biocontrol strain of *Phlebiopsis gigantea* in conifer stumps and its effects on within-species genetic diversity. For. Pathol. 31, 285–295. 10.1046/j.1439-0329.2001.00249.x

[B96] Van Der Wal A. De Boer W. Smant W. Van Veen J. A. (2007). Initial decay of woody fragments in soil is influenced by size, vertical position, nitrogen availability and soil origin. Plant Soil, 301:1–2, 189–201. 10.1007/s11104-007-9437-8

[B97] Van der Wal A. Gunnewiek P. K. de Hollander M. de Boer W. (2017). Fungal diversity and potential tree pathogens in decaying logs and stumps. For. Ecol. Manag. 406, 266–273. 10.1016/j.foreco.2017.08.018

[B98] Van Der Wal A. Ottosson E. De Boer W. (2015). Neglected role of fungal community composition in explaining variation in wood decay rates. Ecology 96, 124–133. 10.1890/14-0242.1 26236897

[B99] Vasaitis R. (2013). Heart rots, sap rots and canker rots. In Infectious Forest Diseases (pp. 197–229). CABI Publishing. 10.1079/9781780640402.0197

[B100] Vasiliauskas R. Larsson E. Larsson K. H. Stenlid J. (2005). Persistence and long-term impact of Rotstop biological control agent on mycodiversity in *Picea abies* stumps. Biol. Control 32, 295–304. 10.1016/j.biocontrol.2004.10.008

[B101] Wickham H. (2009). ggplot2. Springer-Verlag New York. 10.1007/978-0-387-98141-3

[B102] Wisconsin D. N. R. (2021). Red Pine Pocket Mortality. Available online at: https://dnr.wisconsin.gov/topic/foresthealth/redpinepocket (Accessed September 3, 2021).

[B103] Worrall J. J. (1991). Meida for selective isolation of hymenomycetes. Mycologia 83, 241–245. 10.1080/00275514.1991.12026013

[B104] Wright E. S. (2016). Using DECIPHER v2.0 to analyze big biological sequence data in R. R J. 8, 352–359. 10.32614/RJ-2016-025

[B105] Yamashita S. Masuya H. Abe S. Masaki T. Okabe K. (2015). Relationship between the decomposition process of coarse woody debris and fungal community structure as detected by high-throughput sequencing in a deciduous broad-leaved forest in Japan. PLoS ONE 10:e0131510. 10.1371/journal.pone.0131510 26110605 PMC4481346

[B106] Yin M. Wingfield M. J. Zhou X. Linnakoski R. De Beer Z. W. (2019). Taxonomy and phylogeny of the *Leptographium olivaceum* complex (Ophiostomatales, Ascomycota), including descriptions of six new species from China and Europe. MycoKeys 60, 93–123. 10.3897/mycokeys.60.39069 31824211 PMC6898192

[B107] Zaluma A. Sherwood P. Bruna L. Skola U. Gaitnieks T. Rönnberg J. (2021). Control of *Heterobasidion* in Norway spruce stands: the impact of stump cover on efficacy of urea and *Phlebiopsis gigantea* and implications for iorest management. Forests 12:679. 10.3390/f12060679

[B108] Zhao P. S. Guo M. S. Gao G. L. Zhang Y. Ding G. D. Ren Y. Akhtar M. (2020). Community structure and functional group ofroot-associated fungi of Pinus sylvestris var. mongolica across stand ages in the Mu Us Desert. Ecol. Evol. 10, 3032–3042. 10.1002/ece3.6119 32211174 PMC7083681

