# Supplementary material for: Fungal Diversity in Multiple Post-harvest Aged Red Pine Stumps and Their Potential Influence on Heterobasidion Root Rot in Managed Stands Across Minnesota
Source: Front Fungal Biol. 2021 Dec 7;2:782181. doi: 10.3389/ffunb.2021.782181 (PMC10512335; doi:10.3389/ffunb.2021.782181)
Supplement: Supplementary file 1 [file Table_1.docx]

**Supplementary Table 1**

List of fungal taxa obtained and number of isolations from stumps at all sites with native and non-native designating the range of red pine. The numbers in parentheses indicate the years since the stumps were cut and sampling took place. Taxa were identified using ITS sequence comparisons to the best BLASTn match with the NCBI GenBank database. GenBank accession numbers for each taxa are also listed. **following the taxon name indicates that the best blast match was below 97% similarity.

| **Ascomycota** | | | | | | | | | | | |
| --- | --- | --- | --- | --- | --- | --- | --- | --- | --- | --- | --- |
| **Taxa** | **Native (0-1)** | **Non-native (0-1)** | **Native (2-3)** | **Non-native (2-3)** | | **Native (5-6)** | **Non-native (5-6)** | **Native (10-12)** | **Non-native (10-12)** | **Total** | **GenBank Accession #** |
| *Absidia caerulea* |  |  | 1 |  | |  |  |  |  | 1 | OK173730 |
| *Aequabiliella effusa* |  |  |  |  | |  |  | 1 |  | 1 | OK173731 |
| *Alternaria alternata* | 2 | 1 |  | 1 | | 1 |  |  | 2 | 7 | OK173732 |
| *Alternaria arborescens* |  |  |  |  | |  |  |  | 1 | 1 | OK173733 |
| *Alternaria tenuissima* |  | 1 |  |  | | 1 |  |  |  | 2 | OK173734 |
| Ascomycota sp. AJMH-2010** |  |  |  |  | | 1 |  |  |  | 1 | OK173735 |
| *Blastobotrys robertii* |  |  |  |  | |  |  | 2 |  | 2 | OK173736 |
| *Botrytis cinerea* |  |  |  |  | |  |  | 1 |  | 1 | OK173737 |
| *Cadophora fastigiata* |  |  |  | 1 | |  |  |  |  | 1 | OK173738 |
| *Calcarisporium arbuscula* |  |  |  |  | |  | 1 |  |  | 1 | OK173739 |
| *Candida santamariae var. membranifaciens* | 1 |  |  |  | |  |  |  |  | 1 | OK173740 |
| *Chloridium virescens* |  |  |  | 1 | | 1 | 3 |  |  | 5 | OK173741 |
| *Cladobotryum pinarense* |  | 2 |  | 1 | |  |  |  |  | 3 | OK173742 |
| *Cladosporium pseudocladosporioides* | 1 | 1 |  | 1 | |  |  |  |  | 3 | OK173743 |
| *Clonostachys rosea* |  |  |  |  | |  | 1 |  | 1 | 2 | OK173744 |
| *Coniochaeta hoffmannii* |  |  |  |  | |  |  | 2 |  | 2 | OK173745 |
| *Cosmospora berkeleyana* |  |  |  |  | | 1 |  |  |  | 1 | OK173746 |
| *Curvularia coatesiae* |  |  |  |  | |  | 1 |  |  | 1 | OK173747 |
| *Daldinia childiae* |  | 1 |  |  | |  |  |  |  | 1 | OK173748 |
| *Deniquelata* sp. isolate 20SA |  |  |  |  | |  |  |  | 1 | 1 | OK173749 |
| *Didymella bellidis* |  |  |  |  | |  | 1 |  |  | 1 | OK173750 |
| *Didymella* sp. EPU18FB |  |  |  |  | |  |  |  | 1 | 1 | OK173751 |
| *Diplodia sapinea* |  | 3 |  |  | |  |  |  | 2 | 5 | OK173752 |
| *Epicoccum nigrum* | 2 |  |  |  | |  | 1 |  |  | 3 | OK173753 |
| *Fusarium* sp. R194 |  |  |  |  | | 1 |  |  |  | 1 | OK173754 |
| *Grosmannia aurea* |  |  | 3 |  | |  |  |  |  | 3 | OK173755 |
| *Hormonema macrosporum* | 1 |  | 1 |  | | 3 |  |  |  | 5 | OK173756 |
| *Hypocrea lutea* |  |  |  | 1 | |  |  |  |  | 1 | OK173757 |
| *Infundichalara microchona* |  |  |  |  | |  |  | 1 |  | 1 | OK173758 |
| *Karstenula rhodostoma* |  | 1 |  |  | |  |  |  |  | 1 | OK173759 |
| *Kuraishia molischiana* | 1 |  |  |  | |  |  |  |  | 1 | OK173760 |
| *Lecanicillium fusisporum* |  |  |  | 1 | |  | 1 |  |  | 2 | OK173761 |
| *Leptodontidium beauverioides* |  |  |  |  | |  |  |  | 1 | 1 | OK173762 |
| *Leptodontidium camptobactrum* |  |  |  | 1 | |  |  |  |  | 1 | OK173763 |
| *Leptodontidium elatius* |  |  |  |  | |  |  |  | 1 | 1 | OK173764 |
| *Leptographium lundbergii* | 1 |  |  |  | |  |  |  |  | 1 | OK173765 |
| *Leptographium procerum* |  |  |  |  | |  |  |  | 2 | 2 | OK173766 |
| *Leptographium terebrantis* |  |  | 8 |  | |  |  |  |  | 8 | OK173767 |
| *Leptosphaerulina chartarum* |  |  |  | 2 | |  |  |  |  | 2 | OK173768 |
| *Mariannaea elegans* | 1 |  |  | 1 | |  | 2 | 1 | 5 | 10 | OK173769 |
| *Metapochonia bulbillosa* |  | 2 |  | 3 | | 3 | 6 | 1 | 8 | 23 | OK173770 |
| *Metarhizium anisopliae* |  |  |  |  | | 1 |  |  |  | 1 | OK173771 |
| *Meyerozyma guilliermondii* |  |  | 1 |  | |  |  |  |  | 1 | OK173772 |
| *Nakazawaea holstii* |  |  | 1 |  | |  |  |  |  | 1 | OK173773 |
| *Nakazawaea* sp. AK-2019a strain |  |  |  |  | |  |  |  |  |  | OK173774 |
| *Nectria balsamea* |  |  |  |  | |  |  |  | 1 | 1 | OK173775 |
| *Ophiostoma floccosum* | 1 |  |  |  | |  |  |  |  | 1 | OK173776 |
| *Ophiostoma ips* | 1 |  | 2 |  | |  |  |  |  | 2 | OK173777 |
| *Ophiostoma olivaceum* | 1 |  |  |  | |  |  |  |  | 1 | OK173778 |
| *Ophiostoma piceae* |  |  | 1 |  | |  |  |  |  | 1 | OK173779 |
| *Ophiostomatales* sp. SM13-21-21-3 |  |  |  | 3 | | 4 | 4 |  |  | 11 | OK173780 |
| *Paraconiothyrium brasiliense* | 1 |  |  |  | |  | 1 | 1 |  | 3 | OK173781 |
| *Paraconiothyrium fuckelii*** |  |  |  |  | |  | 1 |  |  | 1 | OK173782 |
| *Penicillium bissettii* |  |  | 1 |  | |  |  |  |  | 1 | OK173783 |
| *Penicillium quebecense* |  |  |  |  | |  |  | 1 |  | 1 | OK173784 |
| *Phoma medicaginis* |  |  |  |  | |  |  |  | 1 | 1 | OK173785 |
| *Pilidium lythri* |  |  |  | 1 | |  |  |  |  | 1 | OK173786 |
| *Plectosphaerella cucumerina* |  | 1 |  |  | |  |  |  |  | 1 | OK173787 |
| *Scytalidium album* | 1 | 3 |  | 2 | | 2 |  | 3 | 1 | 12 | OK173788 |
| *Sporothrix* sp. RJ-2014 strain |  | 2 |  | 1 | |  |  |  |  | 3 | OK173789 |
| *Trichoderma atroviride* |  | 1 |  | 1 | |  |  |  |  | 2 | OK173790 |
| *Trichoderma deliquescens* |  |  | 4 |  | |  |  |  |  | 4 | OK173791 |
| *Trichoderma hamatum* |  |  | 1 |  | |  |  |  |  | 1 | OK173792 |
| *Trichoderma harzianum* |  | 2 | 1 | 3 | |  |  |  |  | 6 | OK173793 |
| *Trichoderma koningii* |  |  | 1 |  | |  |  |  |  | 1 | OK173794 |
| *Trichoderma sinuosum* |  |  |  |  | |  |  |  | 1 | 1 | OK173795 |
| *Tympanis abietina* |  | 1 |  |  | |  |  |  |  | 1 | OK173796 |
| *Valsa populina* |  | 2 |  |  | |  |  |  |  | 2 | OK173797 |
| *Xylomelasma* sp. ZK29/08 |  | 1 |  |  | |  | 1 |  |  | 2 | OK173798 |
| **Basidiomycota** | | | | | | | | | | | |
| **Taxa** | **Native (0-1)** | **Non-native (0-1)** | **Native (2-3)** | | **Non-native (2-3)** | **Native (5-6)** | **Non-native (5-6)** | **Native (10-12)** | **Non-native (10-12)** | **Total** |  |
| *Bjerkandera adusta* | 3 |  |  | |  |  |  |  |  | 3 | OK173799 |
| *Cerinosterus* sp. SM 14-21-20-4** |  |  |  | |  | 1 |  |  |  | 1 | OK173800 |
| *Clitopilus* sp. |  | 1 |  | |  |  |  |  |  | 1 | OK173801 |
| *Clitopilus prunulus* |  |  |  | | 1 |  |  |  |  | 1 | OK173802 |
| *Coniophora arida* |  |  |  | | 1 |  |  |  |  | 1 | OK173803 |
| *Coniophora olivacea* |  |  |  | | 1 |  |  |  |  | 1 | OK173804 |
| *Dacrymycetes* sp. 8 ST-2014 |  |  |  | | 1 | 1 |  |  |  | 2 | OK173805 |
| *Emmia lacerata* |  |  |  | |  |  | 1 |  |  | 1 | OK173806 |
| *Irpex lacteus* |  |  |  | |  |  | 4 |  |  | 4 | OK173807 |
| *Meruliopsis* sp. FD-497 |  |  |  | | 1 |  |  |  |  | 1 | OK173808 |
| *Peniophora cinerea* |  |  |  | |  |  | 1 |  | 1 | 2 | OK173809 |
| *Perenniporia corticola* |  |  |  | | 1 |  |  |  |  | 1 | OK173810 |
| *Perenniporia subacida* |  |  |  | |  | 1 |  |  |  | 1 | OK173811 |
| *Phanerochaete* aff. *sordida* I strain FD-463 |  | 1 |  | |  |  |  | 1 |  | 2 | OK173812 |
| *Phanerochaete laevis* |  |  |  | |  | 1 |  |  |  | 1 | OK173813 |
| *Phanerochaete sordida* |  |  |  | |  | 1 |  | 1 |  | 2 | OK173814 |
| *Phanerochaete subserialis* |  |  |  | |  | 1 |  |  |  | 1 | OK173815 |
| *Phlebia chrysocreas* |  |  |  | |  |  | 1 |  |  | 1 | OK173816 |
| *Phlebia livida* |  |  |  | |  |  | 1 |  |  | 1 | OK173817 |
| *Phlebia tremellosa* |  |  |  | | 1 |  | 1 |  |  | 2 | OK173818 |
| *Phlebiopsis gigantea* | 13 | 9 | 14 | | 1 | 1 |  |  |  | 38 | OK173819 |
| *Pholiota spumosa* |  |  | 1 | |  | 1 |  | 3 |  | 5 | OK173820 |
| *Polyporus umbellatus* |  |  | 1 | |  |  |  |  |  | 1 | OK173821 |
| *Scytinostroma* sp. DLL2011-1 |  |  |  | |  | 2 |  |  |  | 2 | OK173822 |
| *Sistotrema brinkmannii* |  |  | 1 | | 1 | 1 | 1 |  |  | 4 | OK173823 |
| *Stereum complicatum* |  |  |  | |  |  | 2 |  |  | 2 | OK173824 |
| *Trametes velutina* |  |  |  | |  |  |  |  | 1 | 1 | OK173825 |
| **Mucormycota** | | | | | | | | | | | |
| **Taxa** | **Native (0-1)** | **Non-native (0-1)** | **Native (2-3)** | | **Non-native (2-3)** | **Native (5-6)** | **Non-native (5-6)** | **Native (10-12)** | **Non-native (10-12)** | **Total** |  |
| *Backusella recurva* |  |  | 1 | |  |  |  |  |  | 1 | OK173826 |
| *Mucor moelleri* |  |  | 1 | |  |  |  |  |  | 1 | OK173827 |
| *Mucor zonatus* |  |  |  | |  | 1 |  |  |  | 1 | OK173828 |
| *Umbelopsis dimorpha* |  |  | 1 | |  |  |  | 1 |  | 2 | OK173829 |
| *Umbelopsis isabellina* | 1 |  | 1 | |  | 3 |  | 6 | 2 | 13 | OK173830 |
| *Umbelopsis nana* |  |  |  | |  |  |  | 2 |  | 2 | OK173831 |
| *Umbelopsis ramanniana* |  | 1 |  | |  |  | 1 | 2 |  | 4 | OK173832 |
| **Mortierellomycota** | | | | | | | | | | | |
| **Taxa** | **Native (0-1)** | **Non-native (0-1)** | **Native (2-3)** | | **Non-native (2-3)** | **Native (5-6)** | **Non-native (5-6)** | **Native (10-12)** | **Non-native (10-12)** | **Total** |  |
| *Mortierella jenkinii* |  |  |  | |  | 1 |  |  |  | 1 | OK173833 |
| *Mortierella* sp. isolate YTM129 |  | 1 |  | |  |  |  | 1 |  | 2 | OK173834 |
| *Mortierella verticillata* |  |  |  | |  | 1 |  |  |  | 1 | OK173835 |
